# A LASSO-based reduced-form CMAQ model for predicting ozone and PM_2.5_ responses to emission changes in South Korea

**DOI:** 10.1371/journal.pone.0347073

**Published:** 2026-09-15

**Authors:** Da-Bin Lee, Hyun-Uk Kang, Gaeun Seo, Jinseok Kim, Bomi Kim, Jung-Hun Woo, Kyu-Baek Hwang

**Affiliations:** 1 Department of Computer Science and Engineering, Graduate School, Soongsil University, Seoul, Republic of Korea; 2 Environmental Planning Institute, Seoul National University, Seoul, Republic of Korea; 3 Department of Advanced Technology Fusion, Konkuk University, Seoul, Republic of Korea; 4 Department of Technology Fusion Engineering, Konkuk University, Seoul, Republic of Korea; 5 Graduate School of Environmental Studies, Seoul National University, Seoul, Republic of Korea; Texas A&M University, UNITED STATES OF AMERICA

## Abstract

Reduced-form models of the Community Multiscale Air Quality Modeling System (CMAQ) enable efficient prediction of air quality responses to emission changes. In this study, we developed a reduced-form CMAQ model based on the least absolute shrinkage and selection operator (LASSO) to approximate CMAQ outputs in high-dimensional settings where the number of emission variables exceeds the number of training samples. CMAQ simulations were generated using 118 emission scenarios covering seven emission sectors across 17 provincial-level administrative regions in South Korea. To account for the bounded nature of pollutant concentrations and to better represent nonlinear responses, an adaptive logit transformation was applied within the LASSO framework. The model was trained on 100 simulations and evaluated on 18 test scenarios, achieving mean root mean square errors of 0.1 ppb for ozone and 0.1 μg/m^3^ for PM_2.5_. The model also identifies a small subset of influential sector–region emission variables, enabling interpretable analysis of emission impacts and supporting the design of targeted emission scenarios. A web-based interface was developed to demonstrate the applicability of the approach, allowing interactive exploration of pollutant responses to emission changes. The reduced-form model enables rapid prediction of air quality responses with substantially lower computational cost than CMAQ simulations.

## Introduction

Air pollution poses significant risks to human health. Major pollutants, such as ozone and PM_2.5_, are associated with premature mortality, particularly from cardiovascular and respiratory diseases [1–5]. Controlling their ambient concentrations is therefore important and requires the regulation of precursor emissions. However, predicting the effects of emission controls is challenging because of diverse meteorological conditions and complex chemical reactions. Chemical transport models [6] are extensively used for this purpose. Among them, the Community Multiscale Air Quality Modeling System (CMAQ) is one of the most widely applied models [7].

CMAQ integrates meteorological and emissions data to simulate hourly pollutant concentrations but requires substantial computational resources. For example, a single control-policy simulation over several months can take multiple days on a typical computing server. As a result, comparing a large number of emission control scenarios using CMAQ alone is impractical.

To address this issue, reduced-form models have been developed to approximate CMAQ responses to emission changes. Among them, response surface models based on multidimensional kriging have been proposed as fast surrogates for CMAQ [8,9], and later improved by integrating principal components analysis with statistical kriging for ozone prediction [10]. Polynomial-based response surface models have also been developed [11]. Subsequent studies extended this framework using deep learning and regression-based input selection methods [12,13]. More recently, various deep learning approaches have been explored for efficient emulation of CMAQ [14–17]. These approaches aim to efficiently approximate CMAQ responses to emission changes using statistical or machine learning models.

However, air quality policy analysis often requires evaluating emission controls across multiple sectors and regions, which can lead to a large number of emission variables. Because CMAQ simulations are computationally expensive, the number of available training scenarios is typically limited. As a result, the number of input variables may exceed the number of training samples, leading to a high-dimensional regression setting that poses challenges for reliable model estimation. Many existing reduced-form approaches have been developed for problems with relatively small numbers of emission variables. Deep learning approaches have also been explored for CMAQ emulation, but they usually require large training datasets and may be less suitable when the number of variables is large relative to the number of training samples.

To address this challenge, we develop a reduced-form CMAQ model for efficiently evaluating emission scenarios for ozone and PM_2.5_. The emission inventory considered in this study includes 119 sector–region emission variables, representing seven emission sectors across 17 provincial-level administrative regions in South Korea. The proposed model is based on the least absolute shrinkage and selection operator (LASSO) [18], a linear regression method that is well suited for problems with many predictors and limited training data [19]. In addition to improving model generalization, LASSO performs variable selection, allowing the identification of sector–region emission variables that most strongly influence ozone and PM_2.5_ concentrations. Because ozone and PM_2.5_ concentrations often exhibit nonlinear relationships with precursor emissions [20–23], we introduce a nonlinear transformation of the response variable within the LASSO framework. The resulting model enables rapid prediction of hourly pollutant concentrations while maintaining the ability to approximate CMAQ outputs. Consequently, the proposed reduced-form model facilitates efficient evaluation of air quality responses to emission changes.

## Materials and methods

### CMAQ simulations

**Fig 1:**
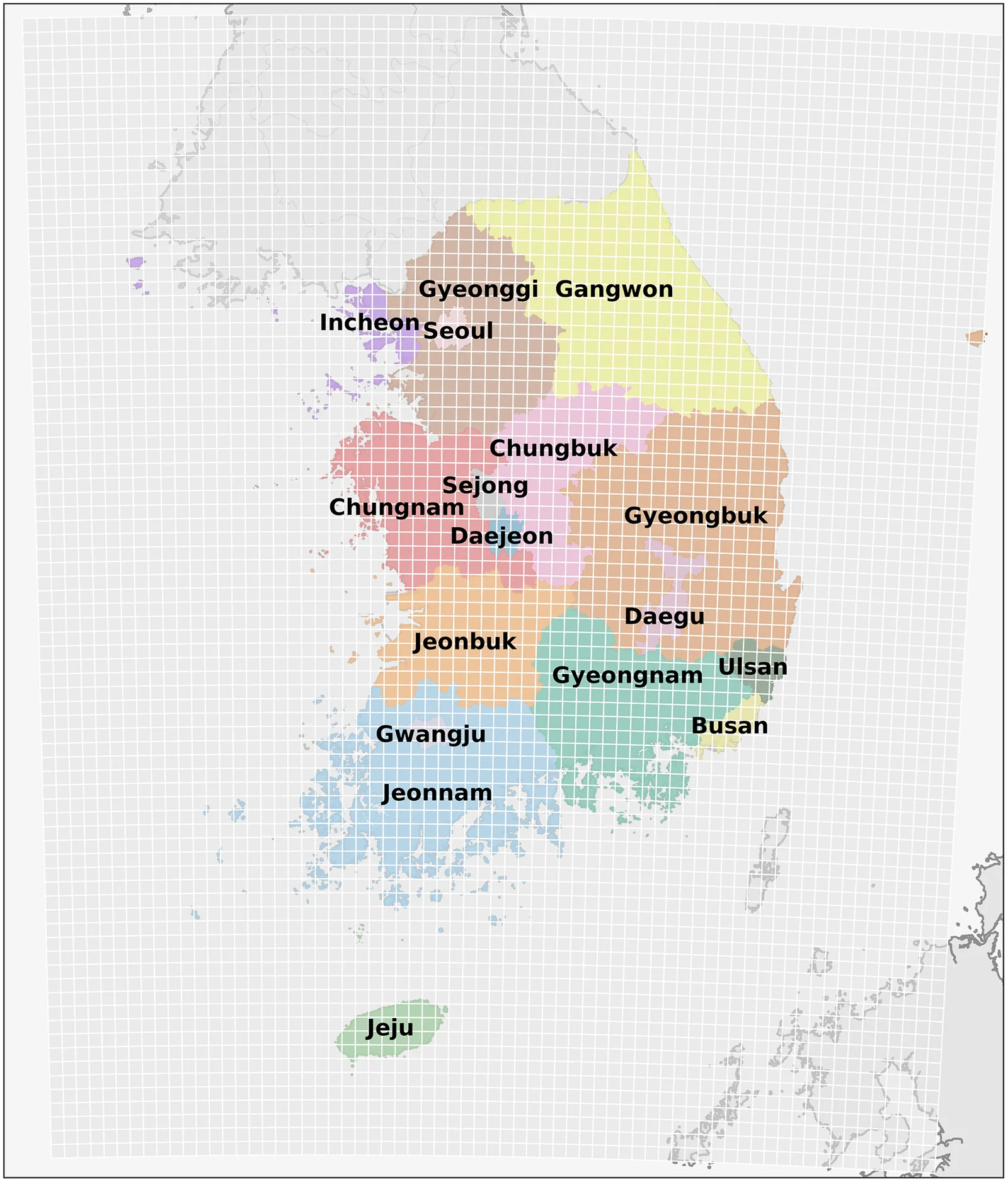
CMAQ simulation domain. The 17 provincial-level administrative regions are shown in different colors, with their names indicated. The map was created by the authors using administrative boundary data from the World Bank Official Boundaries dataset [24] and the Administrative District Boundaries in South Korea (admdongkor) dataset [25], which is derived from Statistics Korea SGIS administrative boundary data [26]. The World Bank Official Boundaries and admdongkor datasets are licensed under the Creative Commons Attribution 4.0 International (CC BY 4.0) License. The underlying SGIS administrative boundary data are released under the Korea Open Government License (KOGL) Type 1, which requires attribution.

The CMAQ simulation domain covered South Korea with 67 × 82 grid cells at a spatial resolution of 9 km × 9 km. Baseline emissions were based on the 2015 national emission inventory provided by the National Institute of Environmental Research, Korea, through an official data request in July 2018. Emissions were reported for the 17 provincial-level administrative regions in South Korea (hereafter referred to as regions; Fig 1). The original inventory consisted of annual total emissions by region, source category, and pollutant. In this study, the original emission source categories were aggregated into seven sectors: Power, Industry, Residential, Solvent, Mobile, Agriculture, and Others (S1 Table).

Each sector-region combination defined one sector–region emission variable, resulting in 119 sector–region emission variables (7 sectors × 17 regions; S2 Table). Emission control scenarios were generated by assigning a scaling factor to each sector-region emission variable. The scaling factor was randomly sampled between 0.5 (50% reduction) and 1.5 (50% increase) and uniformly applied to all pollutant species within the corresponding sector-region combination. Using Latin hypercube sampling [27], 118 scenarios were generated to evenly cover this range.

For each scenario, the annual emission inventory was generated by applying the corresponding scaling factors to the baseline emissions. The inventories were then processed using SMOKE-Asia [28], which converted the annual regional emissions into CMAQ-ready gridded hourly emissions on the 67 × 82 domain at 9 km × 9 km spatial resolution.

Meteorological inputs were generated using the Weather Research and Forecasting model version 3.9.1 [29], with 2017 selected as the reference meteorological year. The 2017 meteorological fields were chosen because they represented typical meteorological conditions and were not associated with unusual weather during the study period.

CMAQ version 4.7.1 [30] was run for four representative months—January, April, July, and October—representing winter, spring, summer, and fall, respectively. A 7-day spin-up period was applied to each simulation to minimize the influence of initial conditions, and outputs from the spin-up period were excluded from all subsequent analyses. The simulations produced hourly ozone and PM_2.5_ concentrations for all grid cells over 123 days (31 days for January, July, and October; 30 days for April). These CMAQ-simulated concentrations were subsequently used as the response variables for training and evaluating the reduced-form models.

To evaluate the baseline CMAQ simulation against observations, CMAQ-simulated concentrations were compared with hourly observations from the AirKorea monitoring network [31] operated by the National Institute of Environmental Research, Korea. Hourly CMAQ concentrations were extracted from the nearest CMAQ grid cell to each AirKorea monitoring station and paired with the corresponding hourly observations. The evaluation was conducted using ozone and PM_2.5_ concentrations from January, April, July, and October of 2017 at monitoring stations across the 17 regions. Model performance was assessed using the correlation coefficient (R), root mean square error (RMSE), and mean bias (MB).

### Machine learning-based reduced-form CMAQ models

**Fig 2:**
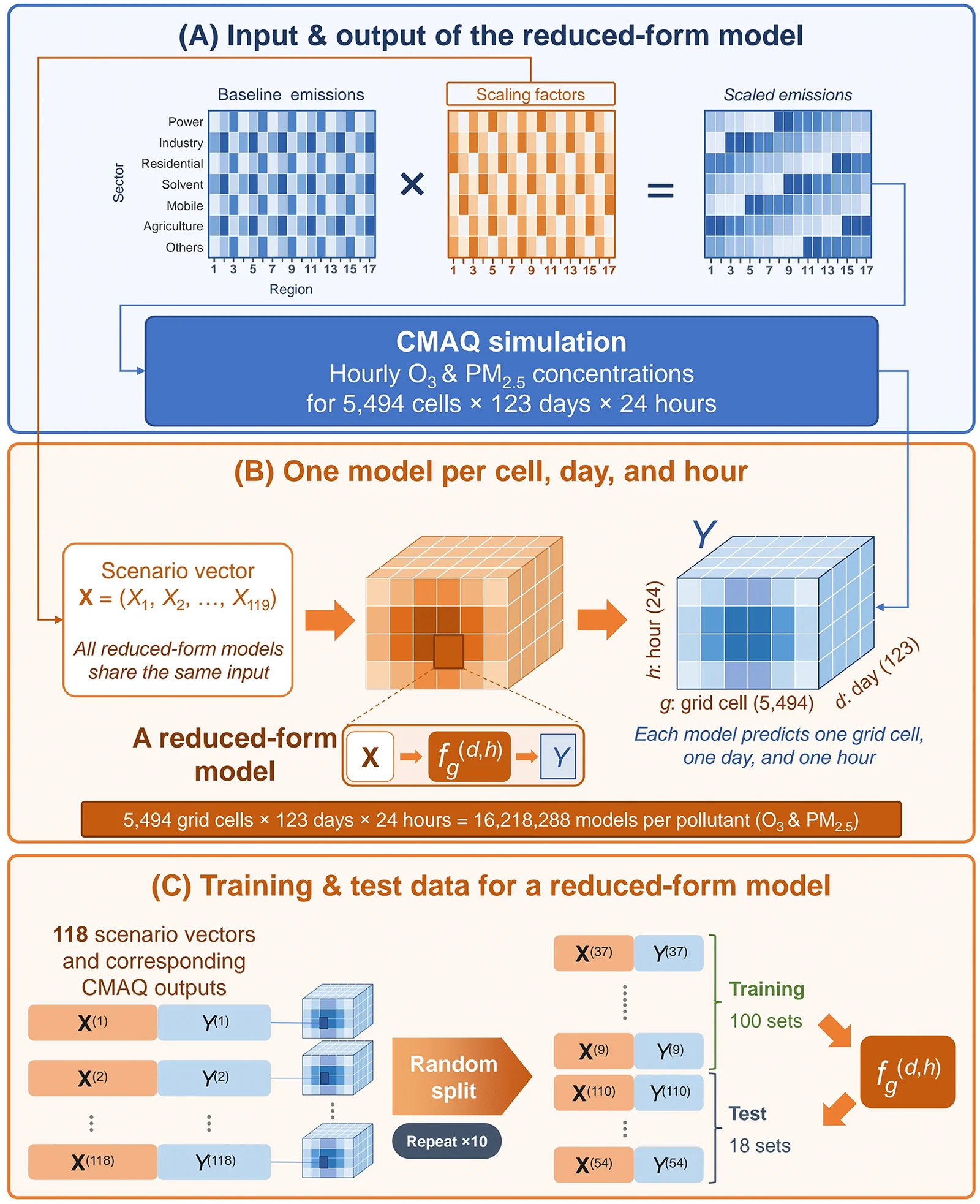
Workflow of the proposed machine learning-based reduced-form CMAQ framework. (A) Generation of CMAQ outputs from emission control scenarios. (B) Independent reduced-form models for individual grid cell-day-hour combinations. (C) Training and performance evaluation of each reduced-form model using repeated random train–test splits.

Fig 2 summarizes the workflow of the proposed machine learning-based reduced-form CMAQ framework. As illustrated in Fig 2A and 2B, the scaling factors applied to the baseline emissions of the 119 sector-region emission variables are assembled into a scenario vector, $𝐗=(X_{\text{1}},X_{\text{2}},\ldots ,X_{\text{1}\text{1}\text{9}})$. The scenario serves as the input to every reduced-form model. Each scenario vector is associated with the corresponding CMAQ-simulated hourly ozone and PM_2.5_ concentrations. Consequently, the reduced-form models are trained directly using pairs of scenario vectors and the corresponding CMAQ simulation results, without requiring a precomputed source–receptor matrix.

As illustrated in Fig 2B, a separate reduced-form model was trained for each combination of grid cell, day, and hour. The reduced-form model for grid cell *g*, day *d*, and hour *h* is denoted by $f_{g}^{\left(d,h\right)}$. The output, denoted by *Y*, represents the CMAQ-simulated ozone or PM_2.5_ concentration for the corresponding grid cell, day, and hour. Because the CMAQ domain contains 5,494 grid cells, 123 days, and 24 hours per day, a total of 16,218,288 independent reduced-form models were trained for each pollutant. This modeling strategy is consistent with previous reduced-form air quality modeling studies, in which grid-level or time-specific response functions were used to represent spatially heterogeneous and temporally varying pollutant responses [9,32–35]. In the present study, this approach was extended to grid–day–hour-specific models to emulate hourly CMAQ concentration responses at the native spatial and temporal resolution of the CMAQ simulations.

Three machine learning methods were evaluated for constructing the reduced-form models: LASSO, support vector regression with a radial basis function kernel (SVR-RBF) [36], and eXtreme Gradient Boosting (XGBoost) [37]. For each method, hyperparameters were optimized using five-fold cross-validation on the training data only. Because hyperparameter optimization was performed using five-fold cross-validation, reduced-form models whose training responses contained five or fewer distinct values (i.e., no more distinct values than the number of folds) were not fitted with machine learning methods. Under such conditions, one or more validation folds may contain only a single distinct response value, making hyperparameter selection unreliable. Instead, the reduced-form model was replaced by a constant predictor equal to the mean of the training responses. Among the three machine learning methods, LASSO was adopted as the proposed reduced-form model based on the comparative evaluation presented in the Results section.

As illustrated in Fig 2C, the dataset for reduced-form model construction consisted of 118 scenario vectors and their corresponding CMAQ-simulated pollutant concentrations. The 118 scenarios were randomly divided into 100 training scenarios and 18 independent test scenarios, and the same train–test split was applied to all reduced-form models. This random partitioning was repeated 10 times to improve the robustness of model evaluation. Model performance was assessed using the RMSE between predicted and CMAQ-simulated concentrations on the independent test scenarios, and the reported RMSE values represent the average over the ten repetitions.

Because ozone and PM_2.5_ concentrations are nonnegative and often exhibit nonlinear responses to emission changes, four response-variable transformations were investigated: untransformed, logarithmic, logit, and the proposed adaptive logit transformation. These transformations are defined as

$$\begin{array}{c} T_{\text{untransformed}}(Y)=Y \end{array}$$, (1)

$$\begin{array}{c} T_{\text{log}}(Y)=\text{log}(Y) \end{array}$$, (2)

$$\begin{array}{c} T_{\text{logit}}=\text{log}\left(\frac{Y}{\text{1}-Y}\right) \end{array}$$, (3)

$$\begin{array}{c} T_{\text{adaptive}}(Y)=\text{log}\left(\frac{Y}{y_{\text{upper}}-Y}\right) \end{array}$$, (4)

For the logit transformation, ozone and PM_2.5_ concentrations were first converted from ppb to ppm and from μg/m^3^ to mg/m^3^, respectively, so that the transformed concentrations satisfied 0<Y<1. In contrast, the proposed adaptive logit transformation replaces the fixed upper bound with


$$\begin{array}{c} y_{\text{upper}}=\text{max}\left({𝐲}_{\text{train}}\right)+\text{SD}\left({𝐲}_{\text{train}}\right) \end{array}$$ (5)

where ${𝐲}_{\text{train}}$ denotes the response values in the corresponding training dataset and SD(·) is the standard deviation. Thus, the upper bound is determined separately for each reduced-form model according to its corresponding training data. For all transformations except the untransformed response, predictions were converted back to the original concentration scale before evaluation. The comparative evaluation of the four response-variable transformations is presented in the Results section.

For inference, a new sector-region scenario vector is supplied to every trained model. The resulting predictions are combined over all grid cells, days, and hours to reconstruct the full grid-level hourly concentration field for ozone or PM_2.5_.

### Coefficient interpretation and influence analysis

As described in the Results section, LASSO was selected as the proposed reduced-form model based on the comparative evaluation of the three machine learning methods. Because LASSO produces sparse and interpretable regression coefficients, these coefficients were further analyzed to quantify the influences of emission sectors and sector–region emission variables. Although each reduced-form model represents pollutant concentrations for only a single grid cell, day, and hour, aggregating the coefficients across the large number of reduced-form models enables the characterization of emission influences averaged over space and time, including overall sectoral influences across South Korea and sector–region influences within individual regions.

**Fig 3:**
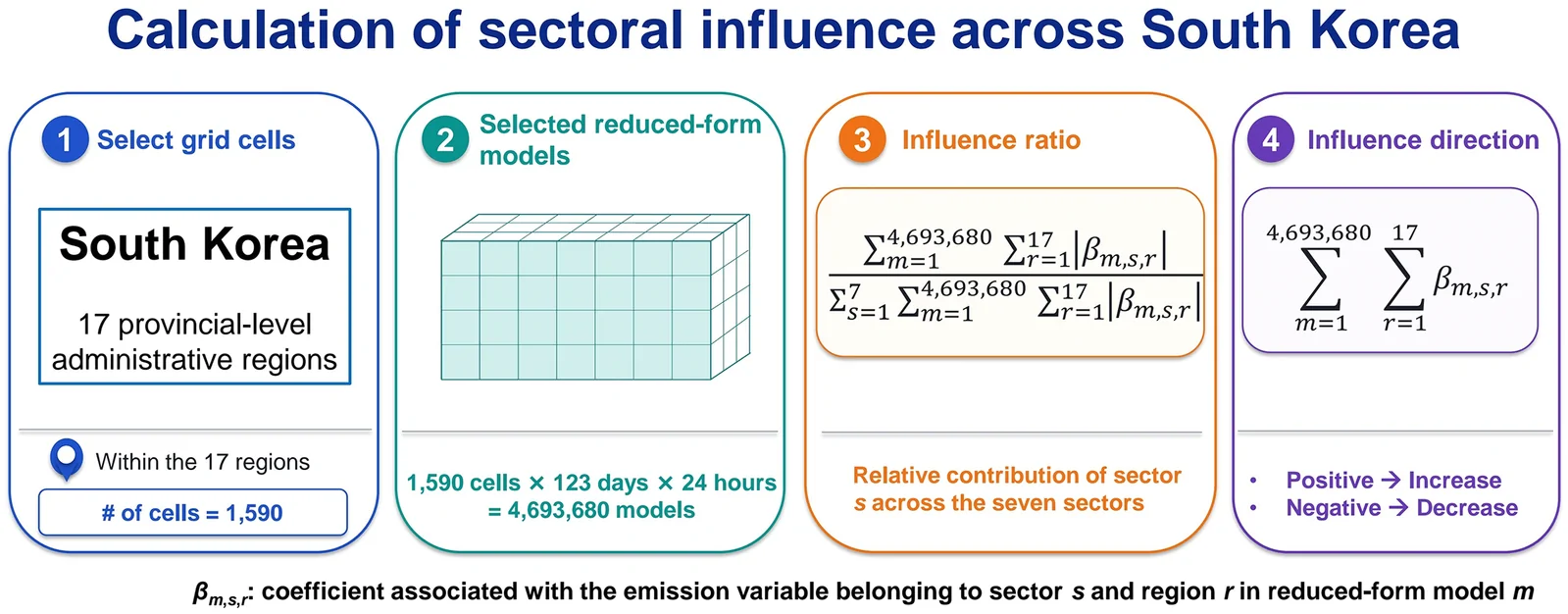
Schematic procedure for calculating sectoral influence ratios and influence directions across South Korea. Reduced-form models corresponding to the 1,590 grid cells within the 17 provincial-level administrative regions were selected, yielding a total of 4,693,680 models (1,590 grid cells × 123 days × 24 hours). Sectoral influence ratios were calculated by aggregating the absolute values of the LASSO coefficients associated with each emission sector across all selected models, whereas influence directions were determined by aggregating the signed coefficients.

To quantify the overall influence of each emission sector across South Korea, the fitted LASSO coefficients were aggregated over all reduced-form models corresponding to the 1,590 grid cells within the 17 regions. The overall procedure is illustrated in Fig 3. A total of 4,693,680 reduced-form models (1,590 grid cells × 123 days × 24 hours) were included in this aggregation. For each emission sector *s*, the absolute values of the coefficients associated with all sector-region emission variables belonging to that sector were summed across all selected reduced form-models:

$$\begin{array}{c} I_{s}=\sum_{m=\text{1}}^{\text{4},\text{6}\text{9}\text{3},\text{6}\text{8}\text{0}}\sum_{r=\text{1}}^{\text{1}\text{7}}\left|\beta_{m,s,r}\right| \end{array}$$, (6)

where $\beta_{m,s,r}$ denotes the coefficient associated with the sector-region emission variable for sector *s* and region *r* in reduced-form model *m*. The sectoral influence ratio for sector *s* was then defined as

$$\begin{array}{c} R_{s}=\frac{I_{s}}{\sum_{s=\text{1}}^{\text{7}}I_{s}} \end{array}$$, (7)

which represents the relative contribution of sector *s* among the seven sectors. The direction of influence was determined from the sign of

$$\begin{array}{c} \sum_{m=\text{1}}^{\text{4},\text{6}\text{9}\text{3},\text{6}\text{8}\text{0}}\sum_{r=\text{1}}^{\text{1}\text{7}}\beta_{m,s,r} \end{array}$$. (8)

A positive value indicates that increasing emissions from the corresponding sector are generally associated with higher pollutant concentrations, whereas a negative value indicates the opposite.

To quantify the influence of individual sector-region emission variables within a specific region, the aggregation procedure described above was applied only to the reduced-form models corresponding to grid cells within the region of interest. The overall procedure is illustrated in S1 Fig. Let *M* denote the number of selected reduced-form models for the corresponding region, which depends on the number of grid cells assigned to that region. For each sector-region emission variable (*s*, *r*), the absolute values of the associated coefficients were summed across the selected reduced-form models:

$$\begin{array}{c} I_{s,r}=\sum_{m=\text{1}}^{M}\left|\beta_{m,s,r}\right| \end{array}$$. (9)

The sector-region influence ratio was then defined as

$$\begin{array}{c} R_{s,r}=\frac{I_{s,r}}{\sum_{s=\text{1}}^{\text{7}}\sum_{r=\text{1}}^{\text{1}\text{7}}I_{s,r}} \end{array}$$. (10)

which represents the relative contribution of the corresponding sector-region emission variable among all the 119 sector-region emission variables. The influence direction was determined in the same manner as for the sectoral influence analysis, using the sign of the aggregated coefficients.

### Emission scenario design

To demonstrate the application of the proposed reduced-form model to emission-control scenario design, coefficient-derived influence ratios were used to identify influential sector–region emission variables for selected receptor regions. For each selected receptor region and pollutant, the five sector–region emission variables with the largest influence ratios were selected as candidate control variables. Starting from the baseline emission setting, two hypothetical scenarios were generated by applying either a 10% reduction or a 10% increase to these variables while all other sector–region emission variables were held at their baseline levels. The trained reduced-form models were then used to predict the resulting changes in ozone and PM_2.5_ concentrations across the simulation domain.

## Results

### Evaluation of the baseline CMAQ simulation

The baseline CMAQ simulation was evaluated against 2017 AirKorea observations. Monthly evaluations yielded R values of 0.45–0.67, RMSE values of 11.4–18.5 ppb, and MB values of −12.8 to −4.7 ppb for ozone. For PM_2.5_, the corresponding R values were 0.69–0.79, RMSE values were 3.54–5.34 μg/m^3^, and MB values were −5.8 to −3.3 μg/m^3^. These results indicate that the baseline CMAQ simulation reasonably reproduced the temporal variability of observed concentrations, particularly for PM_2.5_. However, both pollutants exhibited negative mean biases, indicating a tendency toward underestimation. Because the objective of this study was to develop a reduced-form model that reproduces CMAQ responses to emission perturbations, the baseline CMAQ simulation was considered an appropriate reference for subsequent model development and evaluation.

### Comparison of machine learning methods

Three machine learning methods—LASSO, SVR-RBF, and XGBoost—were compared for constructing the reduced-form CMAQ models. Because the computational costs of SVR-RBF and XGBoost made hourly prediction experiments impractical, this comparison was performed using annual-average pollutant concentrations for each grid cell. All other experimental settings, including the input variables, train-test partitioning, and evaluation procedure, were identical to those used for the hourly prediction experiments.

S3 Table summarizes the prediction accuracy and training time of the three methods. Prediction accuracy was evaluated using RMSE. For ozone, LASSO achieved the lowest RMSE (0.16 ppb), followed by SVR-RBF (0.18 ppb) and XGBoost (0.20 ppb). For PM_2.5_, LASSO again produced the lowest RMSE (0.04 μg/m^3^), whereas SVR-RBF and XGBoost yielded substantially larger errors of 0.11 and 0.12 μg/m^3^, respectively. These results indicate that the linear LASSO model provided more accurate predictions than the kernel-based and tree-based approaches for the present high-dimensional reduced-form modeling problem.

Training time also strongly favored LASSO. For ozone, model training required 7 min for LASSO, compared with 95.1 min for SVR-RBF and 146.7 min for XGBoost. Similar trends were observed for PM_2.5_, with training times of 5 min, 90.8 min, and 1558.8 min, respectively. All experiments were conducted on a workstation equipped with an Intel(R) Xeon(R) CPU E5-1620 v4 at 3.50 GHz with 128 GB RAM.

Considering both prediction accuracy and computational efficiency, LASSO was selected as the proposed reduced-form modeling method for all subsequent hourly prediction experiments.

### Response-variable transformations

The effects of response-variable transformations on prediction accuracy were evaluated using the four transformation methods described in the Materials and methods section: untransformed, logarithmic, logit, and adaptive logit. The results are summarized in S4 Table.

The mean RMSE values were approximately 0.1 for both ozone and PM_2.5_ regardless of the transformation method, indicating comparable average prediction accuracy. In contrast, the maximum RMSE varied substantially among the transformation methods. For ozone, the adaptive logit transformation yielded the smallest maximum RMSE (4.5 ppb), followed by the untransformed response (5.3 ppb), whereas the standard logit and logarithmic transformations produced considerably larger maximum RMSE values of 90.0 and 417.1 ppb, respectively. For PM_2.5_, the adaptive logit transformation again produced the smallest maximum RMSE (5.7 μg/m^3^), followed closely by the standard logit transformation (5.9 μg/m^3^). The logarithmic and untransformed responses yielded maximum RMSE values of 8.9 and 36.3 μg/m^3^, respectively. Here, the maximum RMSE represents the largest RMSE obtained after averaging the RMSE values over the repeated random train–test splits for each grid cell-, day-, and hour-specific reduced-form model.

Overall, the adaptive logit transformation consistently achieved the smallest maximum prediction error for both pollutants while maintaining comparable average prediction accuracy. Therefore, the adaptive logit transformation was adopted for all subsequent LASSO models.

### Prediction accuracy of the proposed reduced-form model

Before evaluating the prediction accuracy of the proposed reduced-form model, we examined how frequently reduced-form models were replaced by constant predictors because of insufficient variation in the training responses. As described in the Materials and methods section, reduced-form models whose training responses contained five or fewer distinct values were replaced by constant predictors equal to the mean of the training responses. The results are summarized in S5 Table. For ozone, this replacement occurred in approximately 10% of all reduced-form models across the entire CMAQ domain, primarily for grid cells outside the 17 regions (e.g., ocean cells), where CMAQ-simulated concentrations exhibited little variation across emission scenarios. Within the 17 regions that constitute the focus of this study, however, only 2,480 cases (proportion = 5.3 × 10^−5^) replaced by constant predictors. For PM_2.5_, such cases were negligible throughout the entire CMAQ domain.

The prediction accuracy of the proposed reduced-form model was evaluated using all reduced-form models, including those replaced by constant predictors because of insufficient variation in the training responses. Under the baseline emission scenario, the average CMAQ-simulated concentrations across all grid cells, days, and hours were 41.5 ppb for ozone and 12.1 μg/m^3^ for PM_2.5_. The corresponding mean RMSE values of the reduced-form model were approximately 0.1 ppb and 0.1 μg/m^3^, respectively, indicating that the prediction errors were small relative to the average CMAQ-simulated concentrations.

**Fig 4:**
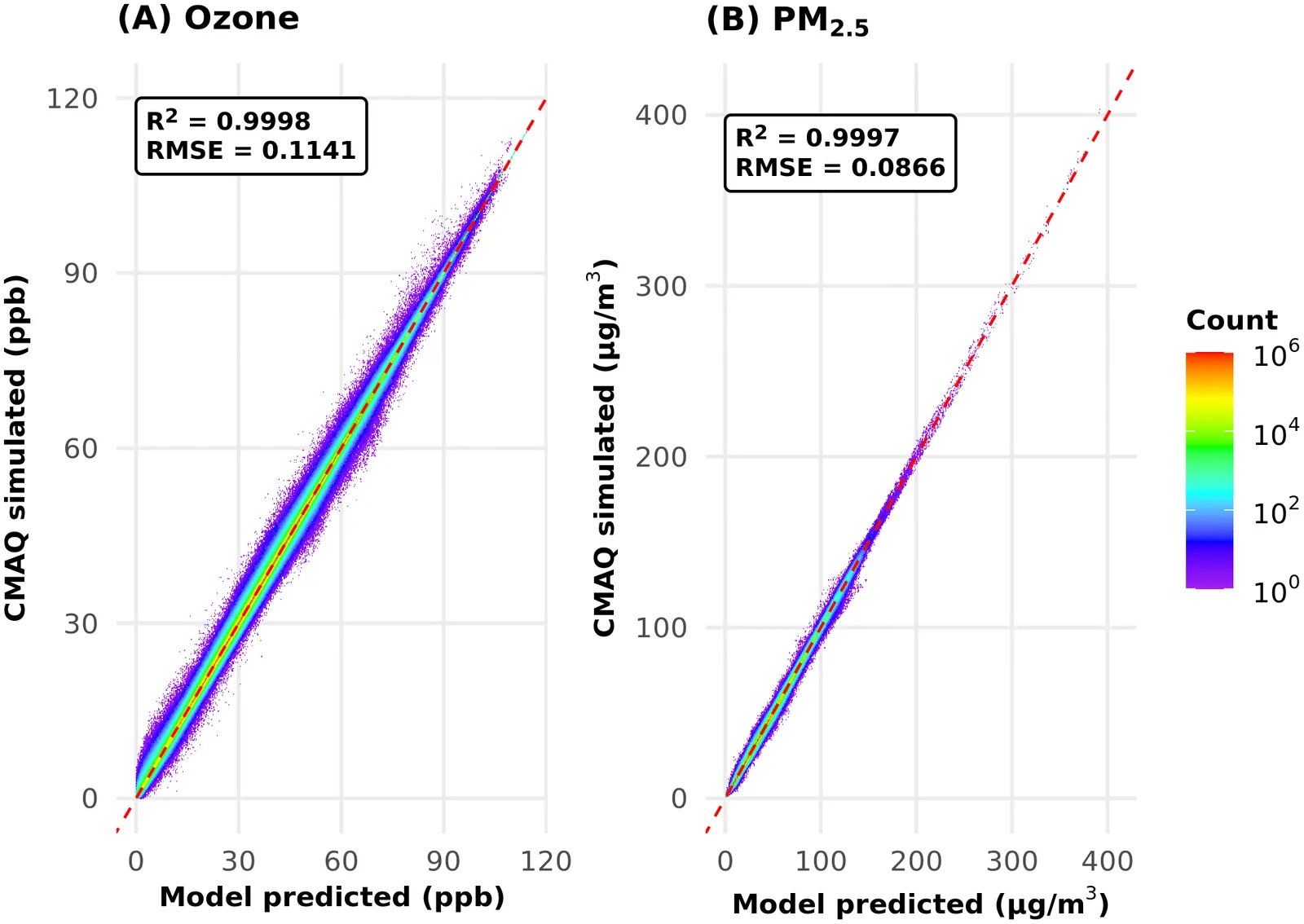
Scatterplots comparing CMAQ-simulated and model-predicted pollutant concentrations. Each point represents a grid cell-hour pair from the test scenarios for (A) ozone and (B) PM_2.5_. Due to the large number of comparisons (18 test scenarios × 10 random train-test splits × 16,218,288 grid cell-hour pairs), 25% of the total data points are shown, while the reported R^2^ and RMSE values are computed using the full dataset.

Fig 4 compares the CMAQ-simulated concentrations to the reduced-form model predictions for the test scenarios. The predictions closely follow the one-to-one line for both ozone (R^2^ = 0.9998) and PM_2.5_ (R^2^ = 0.9997), indicating that the reduced-form model accurately reproduces CMAQ variability across grid cells and time steps.

### Spatial distribution of prediction errors

**Fig 5:**
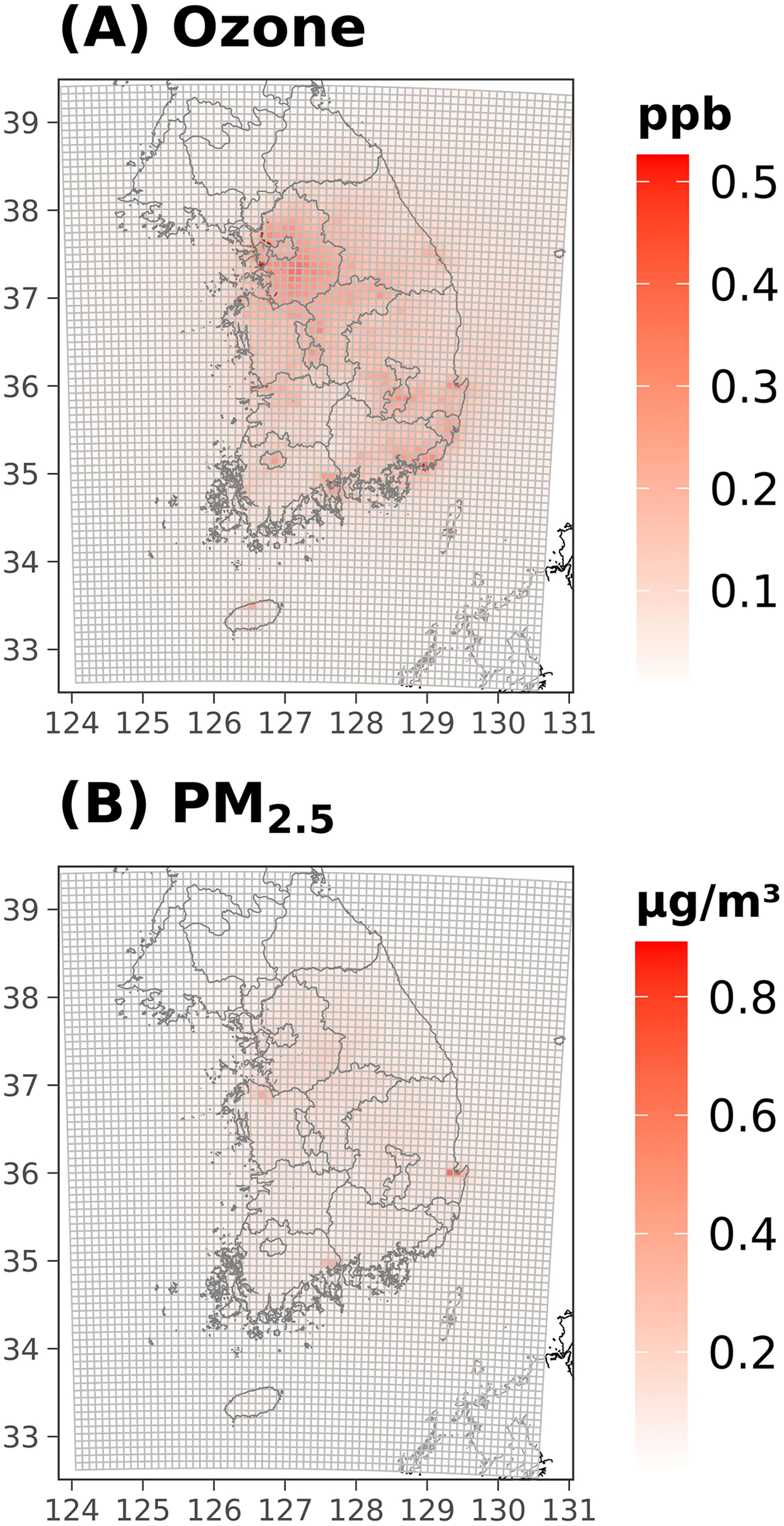
Spatial distribution of prediction errors. RMSE values for each grid cell for (A) ozone and (B) PM_2.5_. The map was created by the authors using administrative boundary data from the World Bank Official Boundaries dataset [24] and the Administrative District Boundaries in South Korea (admdongkor) dataset [25], which is derived from Statistics Korea SGIS administrative boundary data [26]. The World Bank Official Boundaries and admdongkor datasets are licensed under the Creative Commons Attribution 4.0 International (CC BY 4.0) License. The underlying SGIS administrative boundary data are released under the Korea Open Government License (KOGL) Type 1, which requires attribution.

To examine spatial variations in model accuracy, RMSE values were calculated separately for each grid cell by aggregating prediction errors across all test scenarios and hourly predictions. Fig 5 shows the spatial distribution of RMSE for ozone and PM_2.5_ concentrations. Overall, prediction errors remained small across most regions of South Korea. For ozone, RMSE values were typically around 0.1 ppb, with somewhat higher errors (up to about 0.5 ppb) observed in several localized areas. For PM_2.5_, RMSE values were also generally close to 0.1 μg/m^3^. Slightly higher errors (around 0.5 μg/m^3^) were observed in a small number of localized areas (roughly two), and a single localized area showed higher errors reaching approximately 0.8 μg/m^3^.

S2 Fig shows the spatial distribution of the standard deviation of CMAQ-simulated pollutant concentrations, which is similar to the pattern in Fig 5. This suggests that errors were higher in areas with greater concentration variability.

### Interpretation of the reduced-form model

The sparse coefficients produced by LASSO enabled interpretation of the proposed reduced-form model through analyses of sectoral and sector–region influences. Across all reduced-form models, an average of 23.5 variables for ozone and 22.0 variables for PM_2.5_ had nonzero coefficients, indicating that only about 20% of the 119 emission variables were retained in each model. These values represent the average numbers of variables with nonzero coefficients per reduced-form model, calculated across all grid cells, days, hours, and random train-test splits. As shown in S3 Fig, most reduced-form models contained approximately 20–35 nonzero coefficients, although a substantial number of models contained no nonzero coefficients. This indicates that, for some grid cell–day–hour combinations, LASSO selected an intercept-only model, suggesting that the corresponding concentrations were largely insensitive to changes in the emission variables. This sparsity facilitates interpretation by focusing the subsequent analysis on a relatively small subset of influential emission variables.

#### Sectoral influence across the 17 regions.

**Fig 6:**
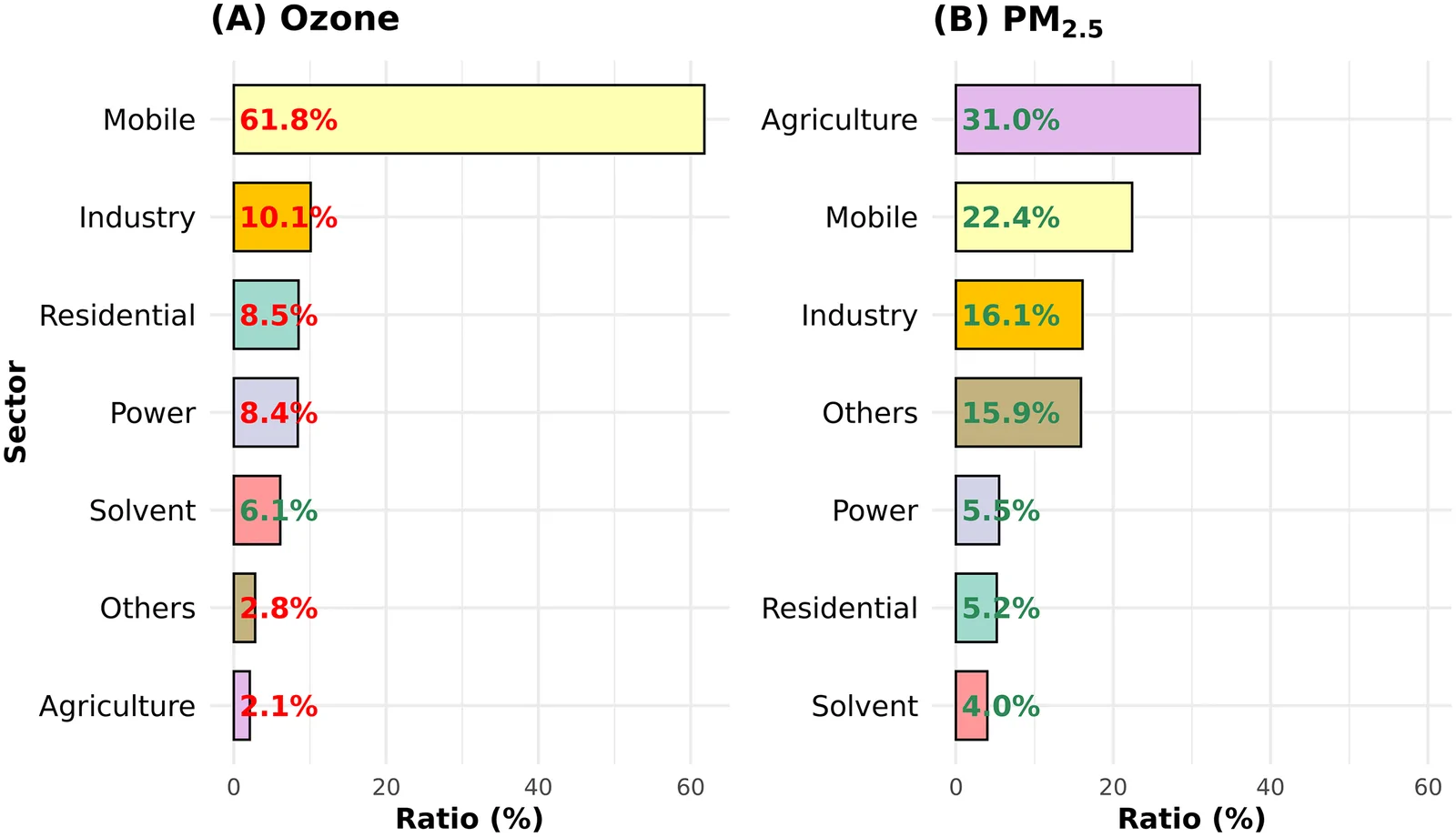
Sectoral influences across the 17 regions. Sectoral influence ratios for (A) ozone and (B) PM_2.5_ are shown. The colors of the ratio values indicate the direction of influence (positive in green, negative in red).

Fig 6 summarizes the sectoral influence ratios and directions across the 17 regions. The sectoral influence ratios were highly consistent across the ten random train–test splits, with standard deviations ranging from only 0.1 to 0.2 percentage points across all sectors, indicating that the aggregated influence estimates were robust to the random train–test partitioning.

For ozone (Fig 6A), the Mobile sector dominated the overall influence, accounting for 61.8% of the total influence ratio. Most sectors exhibited negative influence directions, indicating that increasing emissions generally reduced ozone concentrations. In contrast, the Solvent sector showed a positive influence direction, suggesting that increased solvent emissions were generally associated with higher ozone concentrations.

For PM_2.5_ (Fig 6B), the Agriculture sector had the largest influence ratio (31.0%), followed by the Mobile (22.4%) and Industry (16.1%) sectors. Unlike ozone, all sectors exhibited positive influence directions, indicating that increased emissions were generally associated with higher PM_2.5_ concentrations.

#### Sector-region influence for individual regions.

The five sector–region emission variables with the largest influence ratios for each of the 17 regions are shown in S4 Fig for ozone and S5 Fig for PM_2.5_. Across regions, the combined influence ratios of the five most influential variables ranged from 45.8% to 76.2% for ozone and from 29.1% to 64.9% for PM_2.5_. These results indicate that pollutant responses in each region were largely explained by a relatively small number of sector–region emission variables.

For ozone, the Mobile sector was consistently represented among the five most influential variables across all regions. In many regions, the dominant variables included not only local emissions but also emissions from neighboring regions (Fig 1), reflecting the importance of regional transport.

For PM_2.5_, the influential variables varied more substantially among regions. The Agriculture, Industry, Others, and Mobile sectors were most frequently represented among the five most influential variables, although their relative importance differed among regions. Similar to ozone, influential variables often originated from neighboring regions (Fig 1), indicating that cross-regional transport also contributed to PM_2.5_ responses.

This pattern is illustrated by Seoul. For ozone, the five most influential variables included the Mobile sectors of Incheon and Gyeonggi and the Residential sector of Gyeonggi (S4C Fig). Similarly, for PM_2.5_, the five most influential variables included the Mobile and Others sectors of Gyeonggi as well as the Industry sector of Chungnam (S5C Fig). These examples illustrate that pollutant concentrations within a region are influenced not only by local emissions but also by emissions from surrounding regions, reflecting the regional transport effects represented in the CMAQ simulations.

### Emission scenario evaluation

To demonstrate how the proposed coefficient analysis can be used to design targeted emission scenarios, example scenarios were constructed based on the sector–region variables identified in the previous subsection. Seoul was selected for ozone and Jeju for PM_2.5_, as the five most influential variables accounted for the largest proportions of total influence ratios in these regions (76.2% and 64.9%, respectively; see S4C and S5Q Figs).

For ozone in Seoul, a 10% reduction in the five most influential variables increased the annual mean concentration by 10.0% (12.0 to 13.2 ppb), whereas a 10% increase reduced it by 5.8% (12.0 to 11.3 ppb). For PM_2.5_ in Jeju, a 10% reduction decreased the annual mean concentration by 1.1% (9.3 to 9.2 μg/m^3^), while a 10% increase raised it by 1.1% (9.3 to 9.4 μg/m^3^). These concentration changes were calculated by averaging hourly predicted concentrations over all grid cells within the target administrative region and over all hours of the four representative months (123 days in total).

These examples demonstrate that the proposed approach can identify a small subset of influential sector-region emission variables and use them to construct targeted emission scenarios without exhaustively exploring all 119 sector-region variables.

### Web server for rapid emission scenario evaluation

To facilitate practical use of the proposed model, we developed a web server that enables users to evaluate emission scenarios interactively (http://220.70.0.234:8080/). Users can specify emission changes for the sector–region emission variables, and the server then runs the LASSO-based reduced-form CMAQ model to predict air quality responses.

The server displays the resulting annual mean ozone and PM_2.5_ concentrations on a map, showing grid cell-level values along with the overall mean and range across all grid cells. When a user hovers over a grid cell, the region containing the cell and the predicted annual mean concentration for that cell are shown. When a cell is clicked, the server provides the five most influential sector–region variables for that region along with their influence ratios. These variables provide guidance for designing targeted emission scenarios for the selected region.

In addition to map-based visualization, the server allows users to download hourly concentration files for the four simulated months (see Materials and methods). This enables more detailed analysis beyond the annual mean summaries shown on the map.

The publicly available web server is deployed on a system equipped with an Intel(R) Core(TM) i7-7700 CPU at 4.20 GHz with 44 GB RAM, on which execution of the reduced-form models for both ozone and PM_2.5_ requires less than 30 seconds. This demonstrates that the web server can provide rapid evaluation of emission scenarios while retaining the spatial and temporal detail of CMAQ predictions.

## Discussion

This study developed a LASSO-based reduced-form CMAQ model to efficiently evaluate air quality responses to emission changes across multiple emission sectors and regions. The model achieved high predictive accuracy for both ozone and PM_2.5_, with very small RMSE values relative to the magnitude of the concentrations and near-perfect agreement with CMAQ outputs. These results demonstrate that the proposed approach can successfully approximate CMAQ responses despite the high-dimensional input space and limited number of training scenarios. The adaptive logit transformation further improved the model’s ability to represent nonlinear concentration responses, which are commonly observed for both ozone and PM_2.5_.

A key advantage of the proposed approach is its ability to identify a small subset of influential emission variables from a large set of sector-region combinations. Across all models, only about 20% of the 119 emission variables were selected, indicating substantial sparsity in the relationships between emissions and pollutant concentrations. This sparsity enables meaningful interpretation of model coefficients and facilitates the identification of key emission sources. The sectoral analysis showed that mobile emissions dominated ozone responses, whereas agricultural emissions played the largest role in PM_2.5_ formation. These findings are consistent with the known roles of NO_x_ emissions in ozone chemistry and ammonia emissions in secondary PM_2.5_ formation, supporting the physical plausibility of the model results.

The identified influence ratios should be interpreted within the sampled scenario space rather than as the absolute real-world importance of the corresponding emission sectors. Because the sector–region scaling factors were generated independently using Latin hypercube sampling, the sampled scenarios were designed to isolate the effects of individual emission variables rather than to reproduce realistic correlations among emission changes. This independent sampling reduces confounding among predictors and facilitates interpretation of the estimated influences.

The results also highlight the importance of regional interactions in air quality responses. For both ozone and PM_2.5_, influential variables often included emissions from nearby regions, indicating that pollutant concentrations are affected not only by local emissions but also by regional transport. This suggests that effective air quality management requires coordinated emission control strategies across administrative boundaries rather than region-specific policies alone.

The scenario analysis further demonstrates the utility of the proposed framework for emission scenario design and evaluation. By focusing on a small number of influential variables, the model enables the design of targeted emission scenarios that can produce meaningful changes in pollutant concentrations. For example, in regions where the most influential variables explain a large fraction of the total influence, such as Seoul for ozone and Jeju for PM_2.5_, modifying these variables led to notable changes in concentrations. Specifically, reducing emissions from the most influential variables in Seoul increased ozone concentrations, whereas similar changes resulted in decreases in PM_2.5_ concentrations in Jeju.

The increase in ozone concentration under the emission reduction scenario reflects the predominance of negative aggregated LASSO coefficient sums for the selected variables and is consistent with the CMAQ simulations used for model development. Specifically, we analyzed two of the 118 control scenarios where Mobile sector emissions in Gyeonggi and Seoul increased by 42.7% and 40.1%, and by 37.9% and 41.8%, respectively. The CMAQ simulations showed that annual mean ozone concentrations in Gyeonggi and Seoul decreased by 2.4 ppb and 2.8 ppb. This confirms that even in CMAQ simulations, higher Mobile sector emissions can reduce ozone concentrations. This behavior agrees with the well-established nonlinear response of ozone to precursor emission changes in VOC-limited urban environments, where NO_x_ reductions may increase ozone concentrations [11,38].

Overall, the results illustrate how the model can provide insights into pollutant- and region-specific responses and support the development of more effective and region-specific emission scenarios.

Compared with existing reduced-form approaches, the proposed method is particularly well suited for high-dimensional settings where the number of emission variables is large relative to the number of training simulations. While deep learning models have been increasingly applied for CMAQ emulation, they typically require large training datasets and may be less interpretable. In contrast, the LASSO-based approach used here provides both computational efficiency and interpretability, making it suitable for applications with limited training data and a need for transparent analysis of emission scenarios.

Unlike precursor-oriented reduced-form models such as SA-BPT [39] and APEEP [40], which explicitly represent the effects of individual precursor pollutants, the framework developed in this study is based on sector- and region-specific emission controls. Consequently, precursor species are not included as explicit predictors in the reduced-form models. Instead, precursor effects are implicitly represented through the SMOKE-Asia and CMAQ modeling system used to generate the training data. Sector–region emission scaling factors are first translated into precursor-specific emissions by SMOKE-Asia and then undergo atmospheric transport, chemical transformation, and deposition within CMAQ before the resulting pollutant concentrations are used to train the reduced-form models. Therefore, the proposed framework implicitly captures the net effects of precursor chemistry while directly supporting policy scenario evaluation at the sectoral and regional levels, where emission control measures are typically implemented.

The developed web server was used to demonstrate the practical applicability of the proposed approach by enabling rapid, interactive evaluation of emission scenarios. Through this interface, users can explore air quality responses at the grid-cell level and identify key emission drivers for specific regions, illustrating how the model can support intuitive and efficient scenario design. The ability to generate predictions in under a minute highlights the computational efficiency of the reduced-form model compared with traditional CMAQ simulations, which require substantially longer run times.

Despite the advantages of the proposed approach, several limitations should be noted. First, the model was developed under fixed meteorological conditions and therefore does not account for meteorological variability. The CMAQ simulations used the 2015 baseline emission inventory together with the 2017 meteorological fields. Consequently, although the baseline CMAQ simulation was evaluated against 2017 AirKorea observations, it should be interpreted as a reference simulation for evaluating relative concentration changes in response to emission perturbations under fixed meteorological conditions, rather than as a hindcast intended to reproduce observations for a specific year. The use of a fixed reference meteorological year provides a stable basis for quantifying emission-response relationships and follows the common practice in CMAQ-based emission-control studies, where meteorological conditions are held constant to isolate the effects of emission changes on air quality [41,42]. Nevertheless, the sensitivity of the proposed framework to alternative baseline emission inventories and meteorological years was not examined and should be investigated in future work.

Second, the proposed framework does not explicitly represent temporal dynamics because independent reduced-form models were trained for each grid cell and hour. Although this strategy enables efficient approximation of CMAQ outputs at high spatial and temporal resolution, it does not explicitly exploit temporal dependencies between consecutive time steps.

Third, emission scenarios were generated by independently scaling sector–region emission variables. Consequently, the framework does not account for realistic dependencies among emission changes across sectors and regions. In addition, all precursor species within a given sector–region combination were scaled by the same factor. Therefore, the current framework is well suited for policy-oriented evaluations based on sectoral and regional emission controls but is less appropriate for investigating precursor-specific chemical formation mechanisms, such as separate effects of NO_x_ and VOC emission reductions. Extending the framework to incorporate precursor-specific emission controls represents an important direction for future research.

Future work could extend the proposed framework in several directions. Incorporating meteorological variability would enable application across multiple years and improve the robustness of emission-response analyses under different atmospheric conditions. Integrating temporal dependencies among consecutive time steps may further improve predictive performance while reducing the need to train independent reduced-form models for each hour. Extending the framework to incorporate precursor-specific emission controls would also allow more detailed investigation of ozone and PM_2.5_ formation mechanisms in addition to policy-oriented scenario evaluation. Finally, the proposed framework could be applied to additional pollutants beyond ozone and PM_2.5_ or adapted to other chemical transport models, broadening its applicability to a wider range of air-quality assessment problems. Such extensions would further enhance the utility of the proposed reduced-form framework for efficient, interpretable, and policy-oriented air-quality management.

## Conclusion

This study developed a LASSO-based reduced-form CMAQ model to efficiently evaluate air quality responses to emission changes across multiple sectors and regions. The model achieved high predictive accuracy while substantially reducing computational cost compared with full CMAQ simulations. By identifying a small subset of influential sector–region variables, the proposed approach enables efficient interpretation of emission impacts and the design of targeted emission scenarios. Overall, the results demonstrate that the proposed framework provides a practical and computationally efficient tool for emission scenario evaluation with high-dimensional inputs.

## Supporting information

S1 Fig Schematic procedure for calculating sector–region influence ratios and influence directions within an individual region. The procedure is illustrated using Seoul as an example. Reduced-form models corresponding to the seven grid cells within Seoul were selected, yielding a total of 20,664 models (7 grid cells × 123 days × 24 hours). Sector–region influence ratios were calculated by aggregating the absolute values of the LASSO coefficients associated with each sector–region emission variable across all selected models, whereas influence directions were determined by aggregating the signed coefficients.
(TIF)

S2 Fig Spatial distribution of pollutant concentration variability. Standard deviation of CMAQ-simulated concentrations for each grid cell, for (A) ozone and (B) PM_2.5_. The map was created by the authors using administrative boundary data from the World Bank Official Boundaries dataset [24] and the Administrative District Boundaries in South Korea (admdongkor) dataset [25], which is derived from Statistics Korea SGIS administrative boundary data [26]. The World Bank Official Boundaries and admdongkor datasets are licensed under the Creative Commons Attribution 4.0 International (CC BY 4.0) License. The underlying SGIS administrative boundary data are released under the Korea Open Government License (KOGL) Type 1, which requires attribution.
(TIF)

S3 Fig Distribution of the number of nonzero coefficients per individual LASSO model. Histograms showing the numbers of sector–region emission variables with nonzero coefficients in individual LASSO models for (A) ozone and (B) PM_2.5_.
(DOCX)

S4 Fig Influence ratios of the top five sector-region emission variables for ozone in individual regions. Regions: (A) Incheon, (B) Gyeonggi, (C) Seoul, (D) Gangwon, (E) Chungbuk, (F) Sejong, (G) Daejeon, (H) Chungnam, (I) Gyeongbuk, (J) Daegu, (K) Ulsan, (L) Busan, (M) Gyeongnam, (N) Jeonbuk, (O) Gwangju, (P) Jeonnam, and (Q) Jeju. The colors of the ratio values indicate the direction of influence (positive in green, negative in red). The value in the lower right corner of each plot indicates the sum of the top five influence ratios.
(DOCX)

S5 Fig Influence ratios of the top five sector-region emission variables for PM_2.5_ in individual regions. Regions: (A) Incheon, (B) Gyeonggi, (C) Seoul, (D) Gangwon, (E) Chungbuk, (F) Sejong, (G) Daejeon, (H) Chungnam, (I) Gyeongbuk, (J) Daegu, (K) Ulsan, (L) Busan, (M) Gyeongnam, (N) Jeonbuk, (O) Gwangju, (P) Jeonnam, and (Q) Jeju. The colors of the ratio values indicate the direction of influence (positive in green, negative in red). The value in the lower right corner of each plot indicates the sum of the top five influence ratios.
(DOCX)

S1 Table Grouping of the emission source categories into seven sectors. (DOCX)

S2 Table Provincial-level administrative regions and emission sectors used to define the 119 sector–region emission variables. The reduced-form model input variables consist of all combinations of the 17 provincial-level administrative regions and the seven emission sectors.
(DOCX)

S3 Table Comparison of machine learning methods for constructing reduced-form models. Model performance was evaluated using annual mean concentrations for each grid cell because of the computational cost of SVR-RBF and XGBoost. Hyperparameters were optimized using five-fold cross-validation on the training data.
(DOCX)

S4 Table Summary statistics of prediction errors for ozone and PM_2.5_ under different response-variable transformation. Mean RMSE values were calculated by averaging the RMSE values across all grid cell-, day-, and hour-specific reduced-form models and repeated random train–test splits. Maximum RMSE represents the largest of the RMSE values averaged over the repeated random train–test splits for each grid cell-, day-, and hour-specific reduced-form model.
(DOCX)

S5 Table Reduced-form models replaced by a constant predictor because of insufficient variation in the training responses. A total of 162,182,880 reduced-form models (5,494 grid cells × 123 days × 24 hours × 10 random train-test splits) were evaluated, of which 46,936,800 corresponded to the 17 regions (1,590 grid cells × 123 days × 24 hours × 10 random train-test splits).
(DOCX)
