# Supplementary material for: A LASSO-based reduced-form CMAQ model for predicting ozone and PM2.5 responses to emission changes in South Korea
Source: PLoS One. 2026 Sep 15;21(9):e0347073. doi: 10.1371/journal.pone.0347073 (PMC13577401; doi:10.1371/journal.pone.0347073)
Supplement: Supplementary file 1 [file pone.0347073.s003.docx]

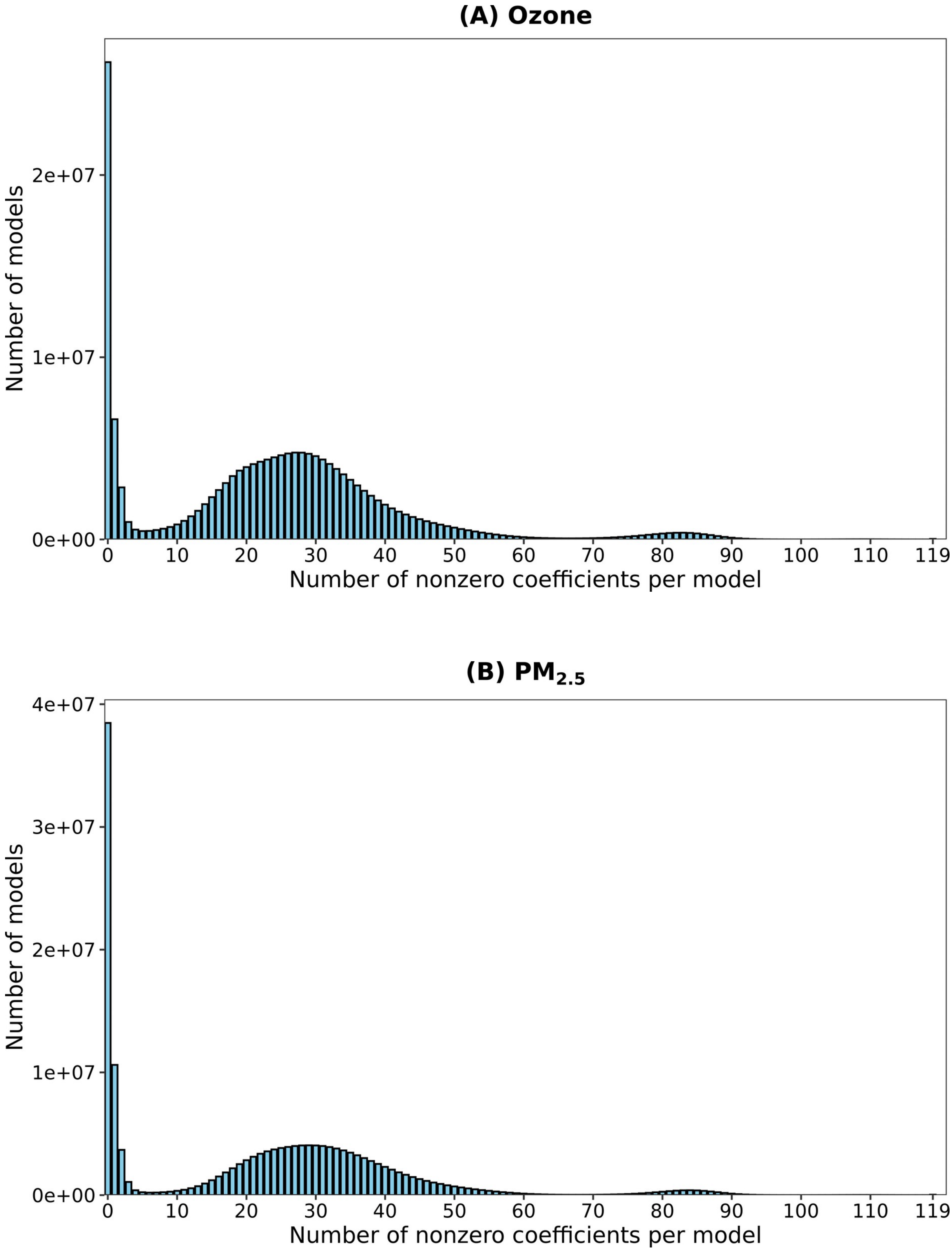
(A) Ozone
Number of models
0e+00
1e+07
2e+07
0
10
20
30
40
50
60
70
80
90
100
110
119
Number of nonzero coefficients per model
(B) $PM_{2.5}$
Number of models
0e+00
1e+07
2e+07
3e+07
4e+07
0
10
20
30
40
50
60
70
80
90
100
110
119
Number of nonzero coefficients per model
