# Supplementary material for: A LASSO-based reduced-form CMAQ model for predicting ozone and PM2.5 responses to emission changes in South Korea
Source: PLoS One. 2026 Sep 15;21(9):e0347073. doi: 10.1371/journal.pone.0347073 (PMC13577401; doi:10.1371/journal.pone.0347073)
Supplement: Supplementary file 2 [file pone.0347073.s004.docx]

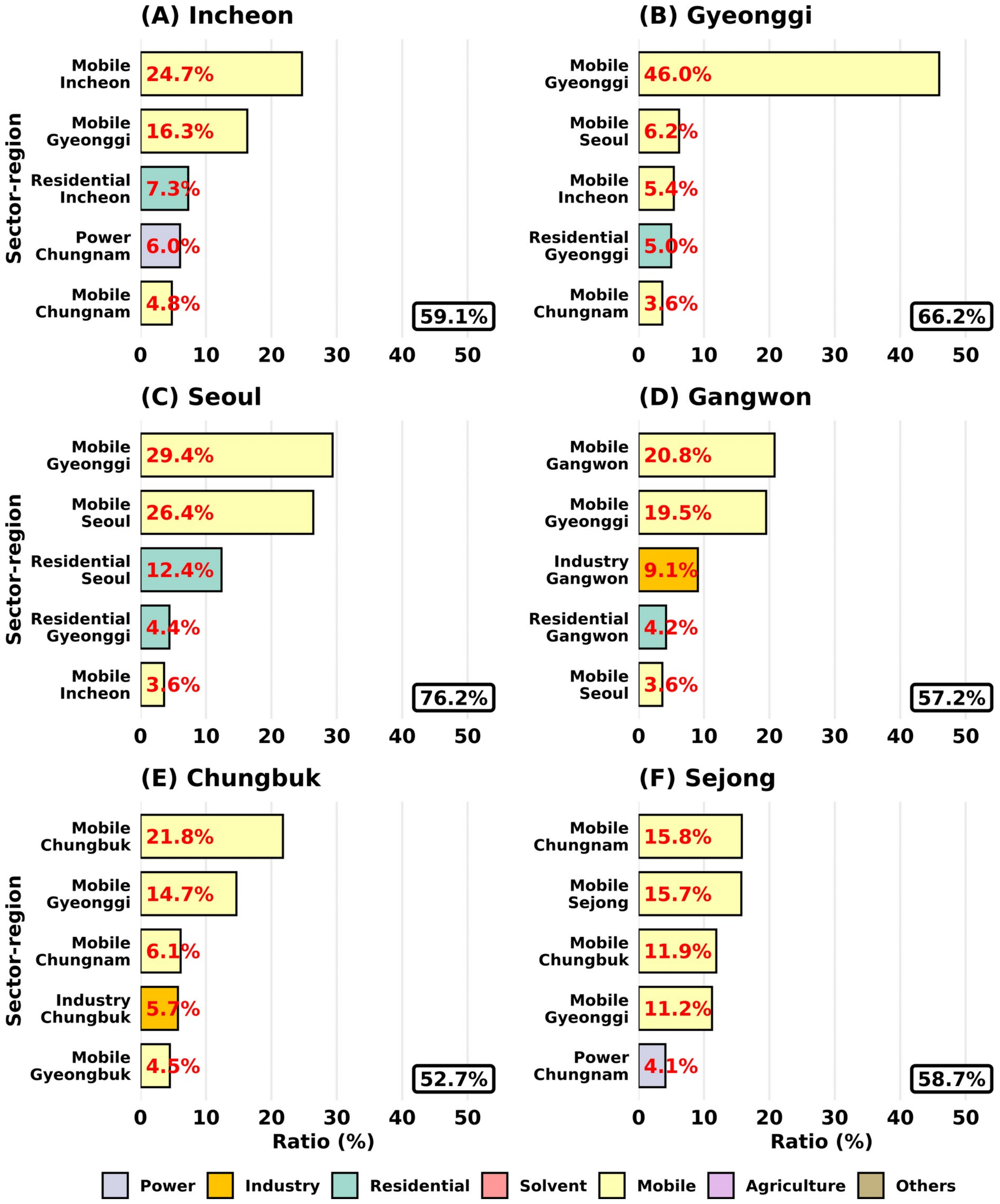
(A) Incheon
Mobile Incheon 24.7%
Mobile Gyeonggi 16.3%
Residential Incheon 7.3%
Power Chungnam 6.0%
Mobile Chungnam 4.8%
59.1%
(B) Gyeonggi
Mobile Gyeonggi 46.0%
Mobile Seoul 6.2%
Mobile Incheon 5.4%
Residential Gyeonggi 5.0%
Mobile Chungnam 3.6%
66.2%
(C) Seoul
Mobile Gyeonggi 29.4%
Mobile Seoul 26.4%
Residential Seoul 12.4%
Residential Gyeonggi 4.4%
Mobile Incheon 3.6%
76.2%
(D) Gangwon
Mobile Gangwon 20.8%
Mobile Gyeonggi 19.5%
Industry Gangwon 9.1%
Residential Gangwon 4.2%
Mobile Seoul 3.6%
57.2%
(E) Chungbuk
Mobile Chungbuk 21.8%
Mobile Gyeonggi 14.7%
Mobile Chungnam 6.1%
Industry Chungbuk 5.7%
Mobile Gyeongbuk 4.5%
52.7%
(F) Sejong
Mobile Chungnam 15.8%
Mobile Sejong 15.7%
Mobile Chungbuk 11.9%
Mobile Gyeonggi 11.2%
Power Chungnam 4.1%
58.7%
Sector-region
Ratio (%)
0 10 20 30 40 50
Power
Industry
Residential
Solvent
Mobile
Agriculture
Others

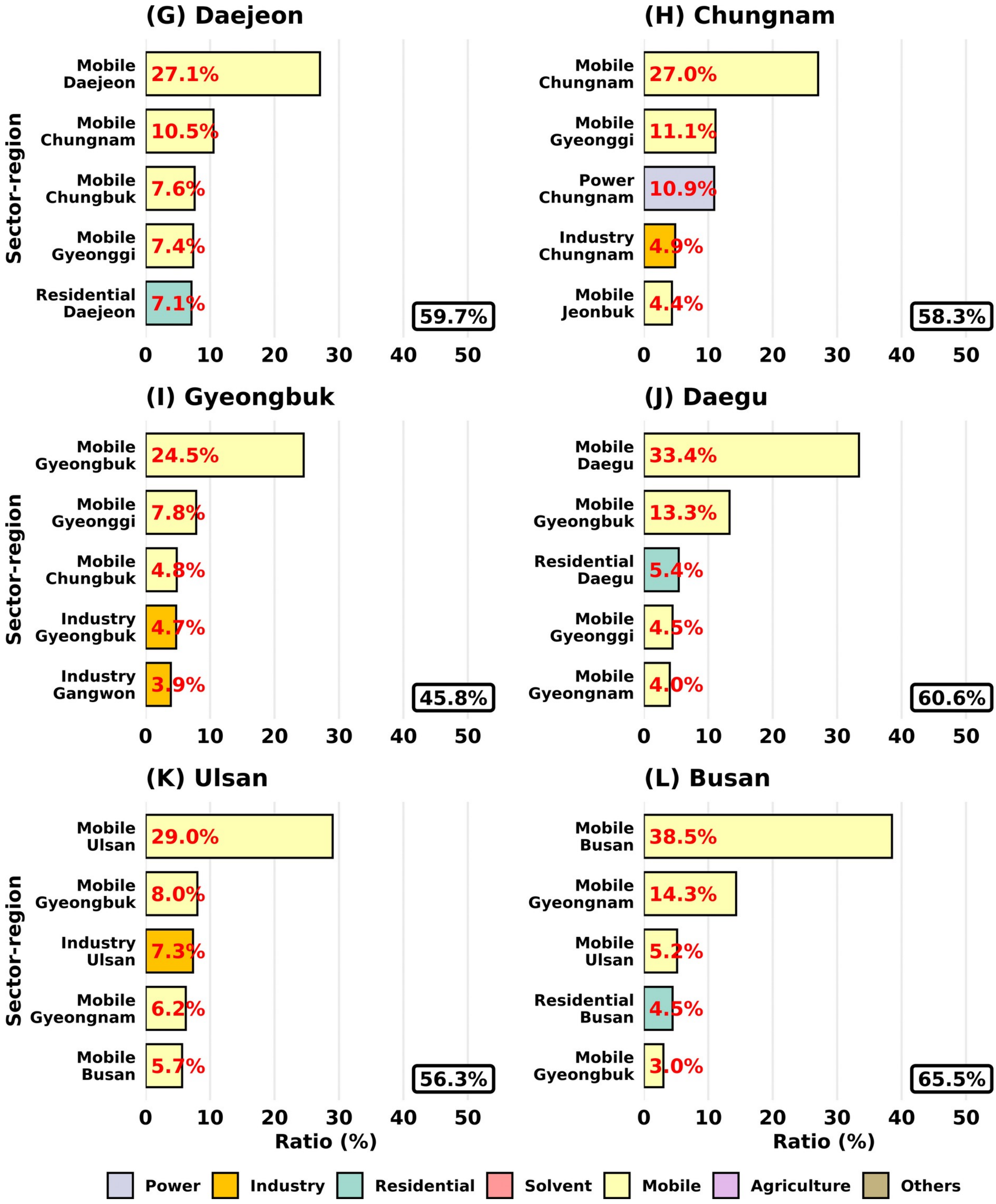

(G) Daejeon
Mobile Daejeon 27.1%
Mobile Chungnam 10.5%
Mobile Chungbuk 7.6%
Mobile Gyeonggi 7.4%
Residential Daejeon 7.1%
59.7%
(H) Chungnam
Mobile Chungnam 27.0%
Mobile Gyeonggi 11.1%
Power Chungnam 10.9%
Industry Chungnam 4.9%
Mobile Jeonbuk 4.4%
58.3%
(I) Gyeongbuk
Mobile Gyeongbuk 24.5%
Mobile Gyeonggi 7.8%
Mobile Chungbuk 4.8%
Industry Gyeongbuk 4.7%
Industry Gangwon 3.9%
45.8%
(J) Daegu
Mobile Daegu 33.4%
Mobile Gyeongbuk 13.3%
Residential Daegu 5.4%
Mobile Gyeonggi 4.5%
Mobile Gyeongnam 4.0%
60.6%
(K) Ulsan
Mobile Ulsan 29.0%
Mobile Gyeongbuk 8.0%
Industry Ulsan 7.3%
Mobile Gyeongnam 6.2%
Mobile Busan 5.7%
56.3%
(L) Busan
Mobile Busan 38.5%
Mobile Gyeongnam 14.3%
Mobile Ulsan 5.2%
Residential Busan 4.5%
Mobile Gyeongbuk 3.0%
65.5%
Sector-region
Ratio (%)
0 10 20 30 40 50
Power
Industry
Residential
Solvent
Mobile
Agriculture
Others

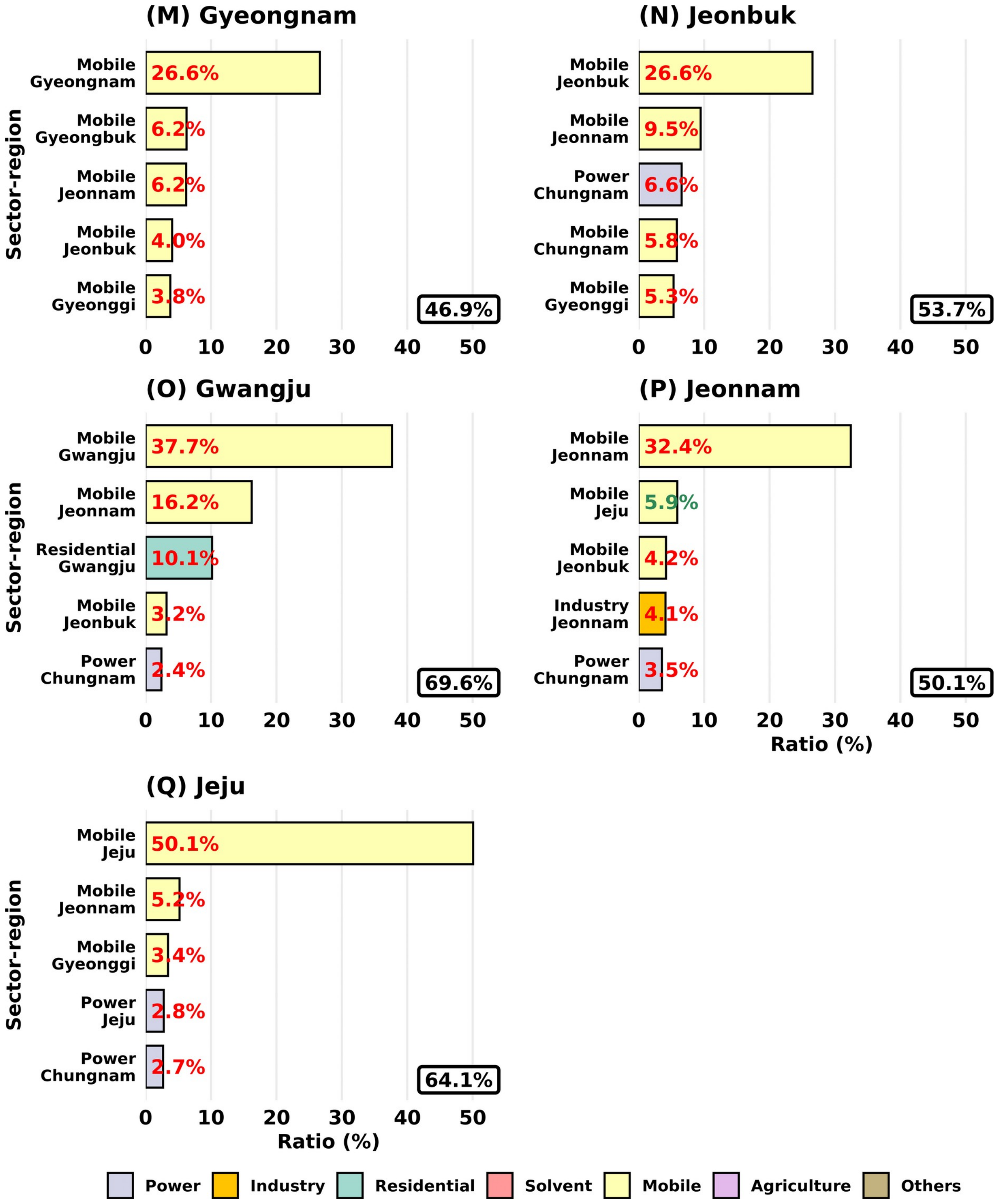
(M) Gyeongnam
Mobile Gyeongnam 26.6%
Mobile Gyeongbuk 6.2%
Mobile Jeonnam 6.2%
Mobile Jeonbuk 4.0%
Mobile Gyeonggi 3.8%
46.9%
(N) Jeonbuk
Mobile Jeonbuk 26.6%
Mobile Jeonnam 9.5%
Power Chungnam 6.6%
Mobile Chungnam 5.8%
Mobile Gyeonggi 5.3%
53.7%
(O) Gwangju
Mobile Gwangju 37.7%
Mobile Jeonnam 16.2%
Residential Gwangju 10.1%
Mobile Jeonbuk 3.2%
Power Chungnam 2.4%
69.6%
(P) Jeonnam
Mobile Jeonnam 32.4%
Mobile Jeju 5.9%
Mobile Jeonbuk 4.2%
Industry Jeonnam 4.1%
Power Chungnam 3.5%
50.1%
(Q) Jeju
Mobile Jeju 50.1%
Mobile Jeonnam 5.2%
Mobile Gyeonggi 3.4%
Power Jeju 2.8%
Power Chungnam 2.7%
64.1%
Sector-region
Ratio (%)
0 10 20 30 40 50
Power
Industry
Residential
Solvent
Mobile
Agriculture
Others
