# Supplementary material for: A LASSO-based reduced-form CMAQ model for predicting ozone and PM2.5 responses to emission changes in South Korea
Source: PLoS One. 2026 Sep 15;21(9):e0347073. doi: 10.1371/journal.pone.0347073 (PMC13577401; doi:10.1371/journal.pone.0347073)
Supplement: Supplementary file 3 [file pone.0347073.s005.docx]

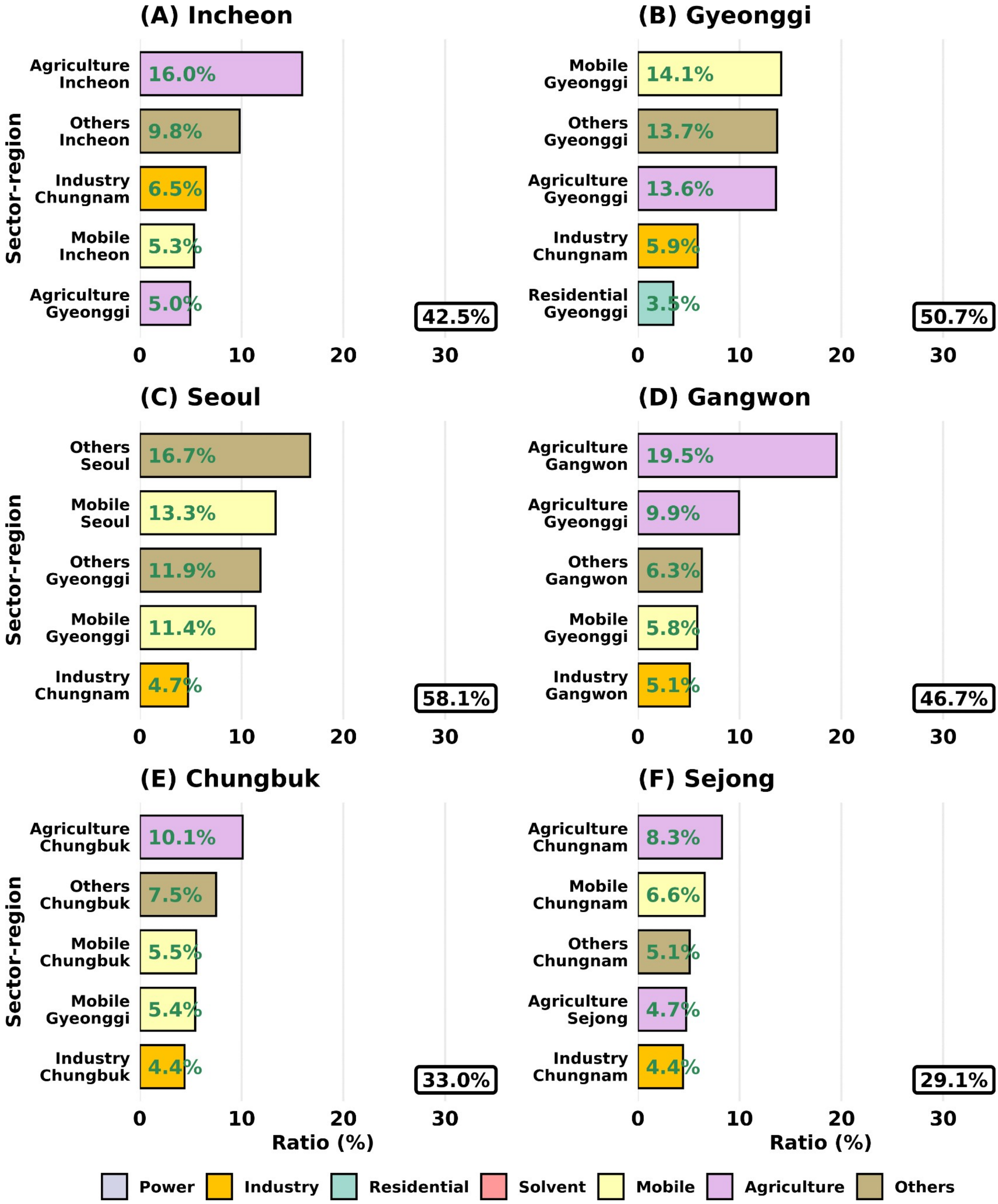

(A) Incheon
Sector-region
Agriculture Incheon
16.0%
Others Incheon
9.8%
Industry Chungnam
6.5%
Mobile Incheon
5.3%
Agriculture Gyeonggi
5.0%
42.5%
(B) Gyeonggi
Mobile Gyeonggi
14.1%
Others Gyeonggi
13.7%
Agriculture Gyeonggi
13.6%
Industry Chungnam
5.9%
Residential Gyeonggi
3.5%
50.7%
(C) Seoul
Others Seoul
16.7%
Mobile Seoul
13.3%
Others Gyeonggi
11.9%
Mobile Gyeonggi
11.4%
Industry Chungnam
4.7%
58.1%
(D) Gangwon
Agriculture Gangwon
19.5%
Agriculture Gyeonggi
9.9%
Others Gangwon
6.3%
Mobile Gyeonggi
5.8%
Industry Gangwon
5.1%
46.7%
(E) Chungbuk
Agriculture Chungbuk
10.1%
Others Chungbuk
7.5%
Mobile Chungbuk
5.5%
Mobile Gyeonggi
5.4%
Industry Chungbuk
4.4%
33.0%
(F) Sejong
Agriculture Chungnam
8.3%
Mobile Chungnam
6.6%
Others Chungnam
5.1%
Agriculture Sejong
4.7%
Industry Chungnam
4.4%
29.1%
0 10 20 30
Ratio (%)
Power
Industry
Residential
Solvent
Mobile
Agriculture
Others

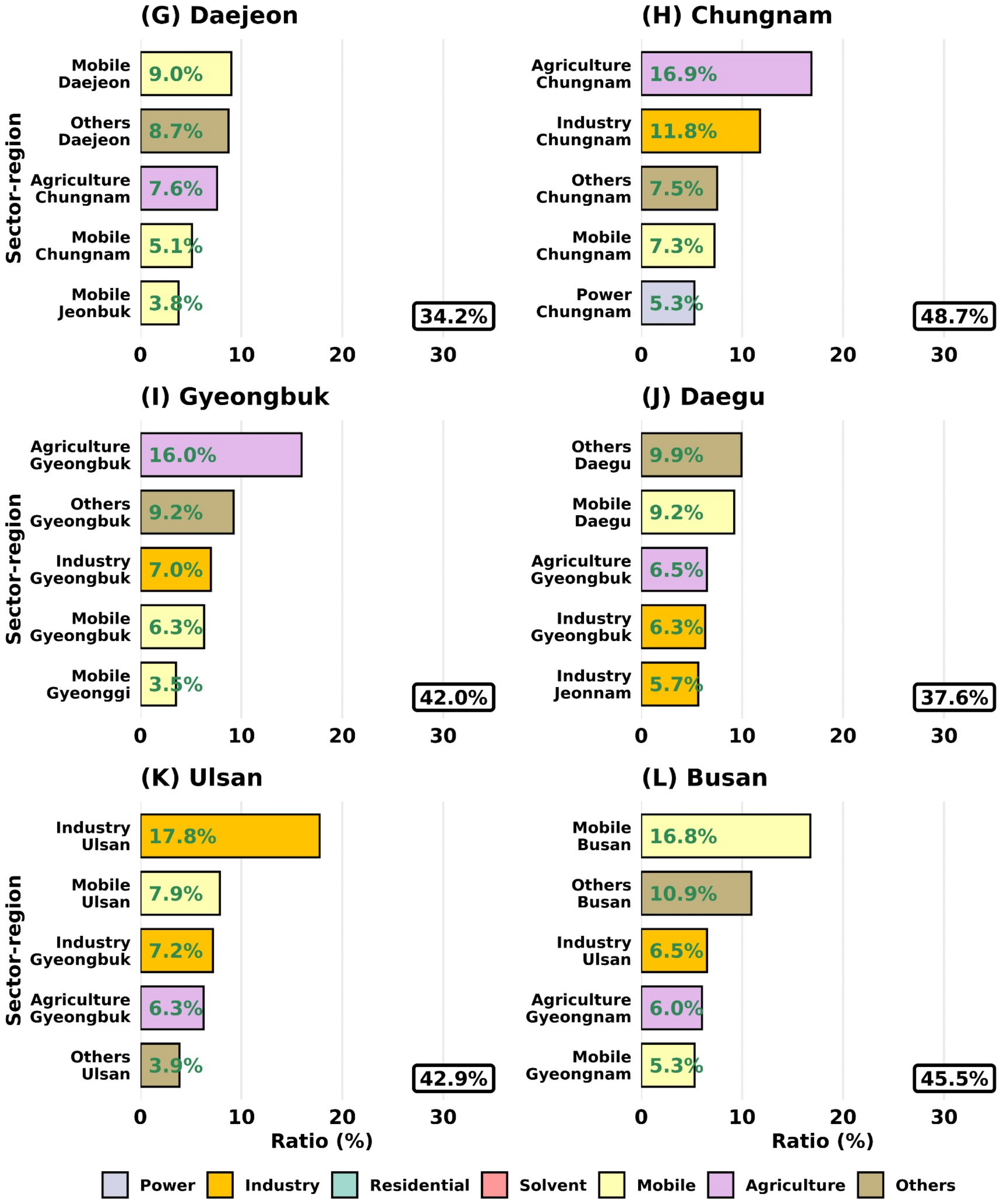
(G) Daejeon
Mobile Daejeon 9.0%
Others Daejeon 8.7%
Agriculture Chungnam 7.6%
Mobile Chungnam 5.1%
Mobile Jeonbuk 3.8%
34.2%
(H) Chungnam
Agriculture Chungnam 16.9%
Industry Chungnam 11.8%
Others Chungnam 7.5%
Mobile Chungnam 7.3%
Power Chungnam 5.3%
48.7%
(I) Gyeongbuk
Agriculture Gyeongbuk 16.0%
Others Gyeongbuk 9.2%
Industry Gyeongbuk 7.0%
Mobile Gyeongbuk 6.3%
Mobile Gyeonggi 3.5%
42.0%
(J) Daegu
Others Daegu 9.9%
Mobile Daegu 9.2%
Agriculture Gyeongbuk 6.5%
Industry Gyeongbuk 6.3%
Industry Jeonnam 5.7%
37.6%
(K) Ulsan
Industry Ulsan 17.8%
Mobile Ulsan 7.9%
Industry Gyeongbuk 7.2%
Agriculture Gyeongbuk 6.3%
Others Ulsan 3.9%
42.9%
(L) Busan
Mobile Busan 16.8%
Others Busan 10.9%
Industry Ulsan 6.5%
Agriculture Gyeongnam 6.0%
Mobile Gyeongnam 5.3%
45.5%
Sector-region
0 10 20 30
Ratio (%)
Power Industry Residential Solvent Mobile Agriculture Others

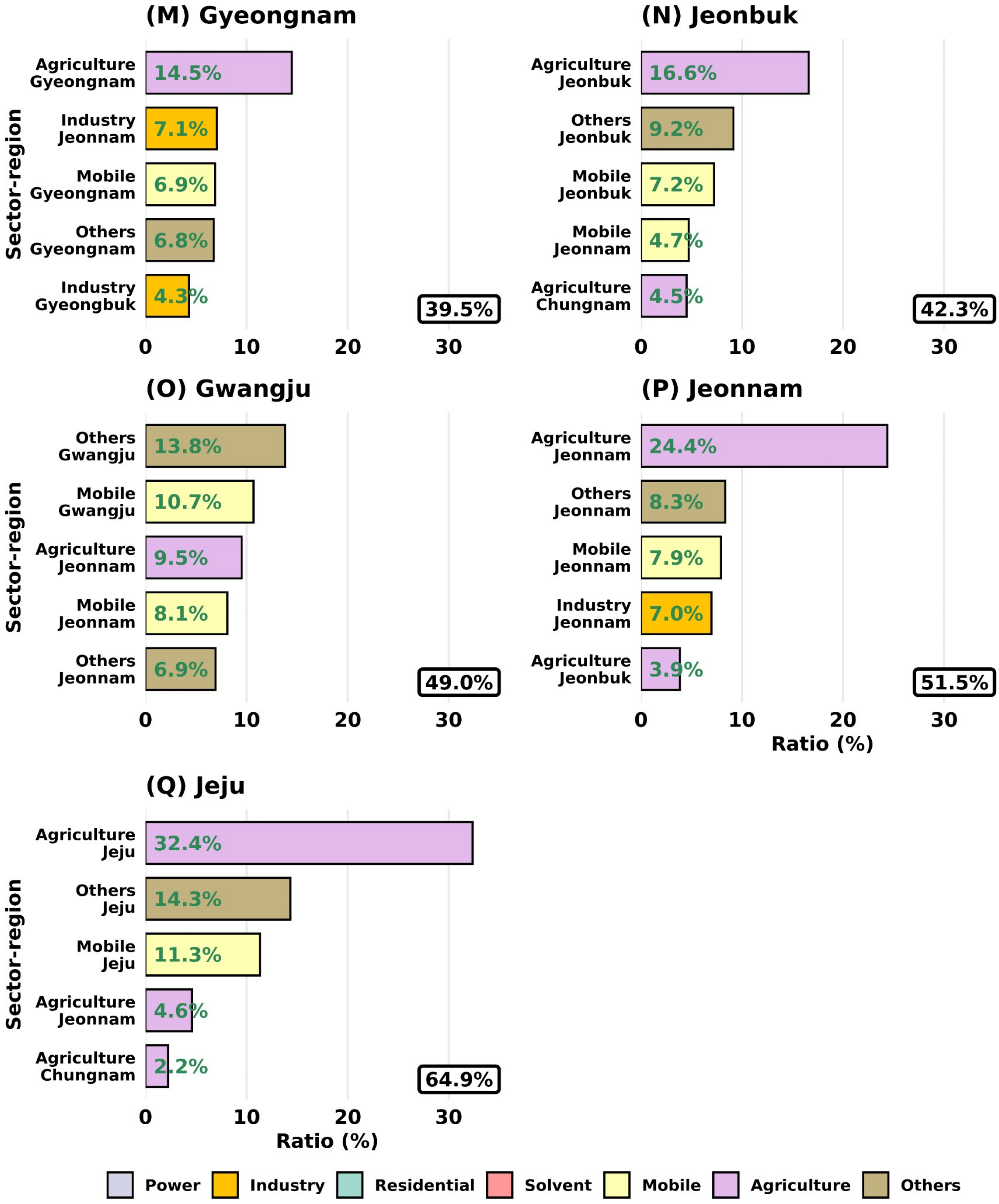
(M) Gyeongnam
Agriculture Gyeongnam 14.5%
Industry Jeonnam 7.1%
Mobile Gyeongnam 6.9%
Others Gyeongnam 6.8%
Industry Gyeongbuk 4.3%
39.5%
(N) Jeonbuk
Agriculture Jeonbuk 16.6%
Others Jeonbuk 9.2%
Mobile Jeonbuk 7.2%
Mobile Jeonnam 4.7%
Agriculture Chungnam 4.5%
42.3%
(O) Gwangju
Others Gwangju 13.8%
Mobile Gwangju 10.7%
Agriculture Jeonnam 9.5%
Mobile Jeonnam 8.1%
Others Jeonnam 6.9%
49.0%
(P) Jeonnam
Agriculture Jeonnam 24.4%
Others Jeonnam 8.3%
Mobile Jeonnam 7.9%
Industry Jeonnam 7.0%
Agriculture Jeonbuk 3.9%
51.5%
(Q) Jeju
Agriculture Jeju 32.4%
Others Jeju 14.3%
Mobile Jeju 11.3%
Agriculture Jeonnam 4.6%
Agriculture Chungnam 2.2%
64.9%
Sector-region
Ratio (%)
0 10 20 30
Power Industry Residential Solvent Mobile Agriculture Others
