# Supplementary material for: A LASSO-based reduced-form CMAQ model for predicting ozone and PM2.5 responses to emission changes in South Korea
Source: PLoS One. 2026 Sep 15;21(9):e0347073. doi: 10.1371/journal.pone.0347073 (PMC13577401; doi:10.1371/journal.pone.0347073)
Supplement: Supplementary file 4 [file pone.0347073.s006.docx]

S1 Table. Grouping of the emission source categories into seven sectors.

| Sector | Emission source category |
| --- | --- |
| Power | - Combustion in energy industries |
| Industry | - Combustion in manufacturing industries  - Industrial processes  - Fugitive emissions from fuel transport and storage |
| Residential | - Non-industrial combustion sources |
| Solvent | - Use of solvents and other products |
| Mobile | - Road transport  - Non-road mobile sources  - Fugitive dust (re-entrained road dust; fugitive dust from unpaved roads) |
| Agriculture | - Agriculture  - Fugitive dust (agricultural activities; livestock activities) |
| Others | - Waste treatment and disposal  - Other area sources  - Biomass burning  - Fugitive dust (construction activities; vacant land; loading and storage operations; waste treatment and disposal) |
