# Supplementary material for: A LASSO-based reduced-form CMAQ model for predicting ozone and PM2.5 responses to emission changes in South Korea
Source: PLoS One. 2026 Sep 15;21(9):e0347073. doi: 10.1371/journal.pone.0347073 (PMC13577401; doi:10.1371/journal.pone.0347073)
Supplement: Supplementary file 5 [file pone.0347073.s007.docx]

S2 Table. Provincial-level administrative regions and emission sectors used to define the 119 sector–region emission variables. The reduced-form model input variables consist of all combinations of the 17 provincial-level administrative regions and the seven emission sectors.

| Provincial-level administrative regions (17) | Emission sectors (7) |
| --- | --- |
| Incheon | Power |
| Gyeonggi | Industry |
| Seoul | Residential |
| Gangwon | Solvent |
| Chungbuk | Mobile |
| Sejong | Agriculture |
| Daejeon | Others |
| Chungnam |  |
| Gyeongbuk |  |
| Daegu |  |
| Ulsan |  |
| Busan |  |
| Gyeongnam |  |
| Jeonbuk |  |
| Gwangju |  |
| Jeonnam |  |
| Jeju |  |
