# Supplementary material for: A LASSO-based reduced-form CMAQ model for predicting ozone and PM2.5 responses to emission changes in South Korea
Source: PLoS One. 2026 Sep 15;21(9):e0347073. doi: 10.1371/journal.pone.0347073 (PMC13577401; doi:10.1371/journal.pone.0347073)
Supplement: Supplementary file 6 [file pone.0347073.s008.docx]

**S3 Table. Comparison of machine learning methods for constructing reduced-form models.** Model performance was evaluated using annual mean concentrations for each grid cell because of the computational cost of SVR-RBF and XGBoost. Hyperparameters were optimized using five-fold cross-validation on the training data.

| Pollutant | Metric | LASSO | SVR-RBF | XGBoost |
| --- | --- | --- | --- | --- |
| Ozone | RMSE (ppb) | 0.16 | 0.18 | 0.20 |
|  | Training time (min) | 7.0 | 95.1 | 146.7 |
| PM_2.5_ | RMSE (μg/m^3^) | 0.04 | 0.11 | 0.12 |
|  | Training time (min) | 5.0 | 90.8 | 1558.8 |
