# Supplementary material for: A LASSO-based reduced-form CMAQ model for predicting ozone and PM2.5 responses to emission changes in South Korea
Source: PLoS One. 2026 Sep 15;21(9):e0347073. doi: 10.1371/journal.pone.0347073 (PMC13577401; doi:10.1371/journal.pone.0347073)
Supplement: Supplementary file 7 [file pone.0347073.s009.docx]

**S4 Table. Summary statistics of prediction errors for ozone and PM_2.5_ under different response-variable transformation.** Mean RMSE values were calculated by averaging the RMSE values across all grid cell-, day-, and hour-specific reduced-form models and repeated random train–test splits. Maximum RMSE represents the largest of the RMSE values averaged over the repeated random train–test splits for each grid cell-, day-, and hour-specific reduced-form model.

| Transformation  Method | Ozone RMSE (ppb) | | PM_2.5_ RMSE (μg/m^3^) | |
| --- | --- | --- | --- | --- |
|  | **Mean** | **Max** | **Mean** | **Max** |
| Untransformed | 0.1 | 5.3 | 0.1 | 36.3 |
| Log | 0.1 | 417.1 | 0.1 | 8.9 |
| Logit | 0.1 | 90.0 | 0.1 | 5.9 |
| Adaptive logit | 0.1 | 4.5 | 0.1 | 5.7 |
