# Supplementary material for: A LASSO-based reduced-form CMAQ model for predicting ozone and PM2.5 responses to emission changes in South Korea
Source: PLoS One. 2026 Sep 15;21(9):e0347073. doi: 10.1371/journal.pone.0347073 (PMC13577401; doi:10.1371/journal.pone.0347073)
Supplement: Supplementary file 8 [file pone.0347073.s010.docx]

S5 Table. Reduced-form models replaced by a constant predictor because of insufficient variation in the training responses. A total of 162,182,880 reduced-form models (5,494 grid cells × 123 days × 24 hours × 10 random train-test splits) were evaluated, of which 46,936,800 corresponded to the 17 regions (1,590 grid cells × 123 days × 24 hours × 10 random train-test splits).

| Cell type | Ozone | | PM_2.5_ | |
| --- | --- | --- | --- | --- |
|  | Number | Proportion | Number | Proportion |
| Region | 2,480 | $5.3\times{10}^{-5}$ | 0 | 0.0 |
| Non-region | 12,210,576 | 0.1 | 10 | $8.7\times{10}^{-8}$ |
| All | 12,213,056 | 0.1 | 10 | $6.2\times{10}^{-8}$ |
