# Supplementary material for: A LASSO-based reduced-form CMAQ model for predicting ozone and PM2.5 responses to emission changes in South Korea
Source: PLoS One. 2026 Sep 15;21(9):e0347073. doi: 10.1371/journal.pone.0347073 (PMC13577401; doi:10.1371/journal.pone.0347073)
Supplement: Supplementary file 9 [file pone.0347073.s012.docx]

PONE-D-26-15138

A LASSO-based reduced-form CMAQ model for predicting ozone and PM2.5 responses to emission changes in South Korea

PLOS One

Dear Dr. Hwang,

Thank you for submitting your manuscript to PLOS ONE. After careful consideration, we feel that it has merit but does not fully meet PLOS ONE’s publication criteria as it currently stands. Therefore, we invite you to submit a revised version of the manuscript that addresses the points raised during the review process.

Please submit your revised manuscript by Jul 11 2026 11:59PM. If you will need more time than this to complete your revisions, please reply to this message or contact the journal office at plosone@plos.org. When you're ready to submit your revision, log on to <https://www.editorialmanager.com/pone/> and select the 'Submissions Needing Revision' folder to locate your manuscript file.

Please include the following items when submitting your revised manuscript:

- A letter that responds to each point raised by the academic editor and reviewer(s). You should upload this letter as a separate file labeled 'Response to Reviewers'.
- A marked-up copy of your manuscript that highlights changes made to the original version. You should upload this as a separate file labeled 'Revised Manuscript with Track Changes'.
- An unmarked version of your revised paper without tracked changes. You should upload this as a separate file labeled 'Manuscript'.

If you would like to make changes to your financial disclosure, please include your updated statement in your cover letter. Guidelines for resubmitting your figure files are available below the reviewer comments at the end of this letter.

If applicable, we recommend that you deposit your laboratory protocols in [protocols.io](http://protocols.io/) to enhance the reproducibility of your results. Protocols.io assigns your protocol its own identifier (DOI) so that it can be cited independently in the future. For instructions see: [https://journals.plos.org/plosone/s/submission-guidelines#loc-laboratory-protocols](https://track.editorialmanager.com/CL0/https:%2F%2Fjournals.plos.org%2Fplosone%2Fs%2Fsubmission-guidelines%23loc-laboratory-protocols/1/010f019e68322324-6834da5b-9fd9-4735-8b74-220838c16dba-000000/y5QYxThmt7V-zIDLjL-odHvBFSo7lUMo2q8kyeUEPtE=258). Additionally, PLOS ONE offers an option for publishing peer-reviewed Lab Protocol articles, which describe protocols hosted on [protocols.io](http://protocols.io/). Read more information on sharing protocols at [https://plos.org/protocols?utm_medium=editorial-email&utm_source=authorletters&utm_campaign=protocols](https://track.editorialmanager.com/CL0/https:%2F%2Fplos.org%2Fprotocols%3Futm_medium=editorial-email%26utm_source=authorletters%26utm_campaign=protocols/1/010f019e68322324-6834da5b-9fd9-4735-8b74-220838c16dba-000000/sXhxiiNNWJly4ZcYV4adA5dv8711v6qn4WBK6LuXiiE=258).

As the corresponding author, your ORCID iD is verified in the submission system and will appear in the published article. PLOS supports the use of ORCID, and we encourage all coauthors to register for an ORCID iD and use it as well. Please encourage your coauthors to verify their ORCID iD within the submission system before final acceptance, as unverified ORCID iDs will not appear in the published article. *Only* the individual author can complete the verification step; PLOS staff *cannot* verify ORCID iDs on behalf of authors.

We look forward to receiving your revised manuscript.

Kind regards,

Yangyang Xu
Academic Editor
PLOS One

Journal Requirements:

When submitting your revision, we need you to address these additional requirements.

1. Please ensure that your manuscript meets PLOS ONE's style requirements, including those for file naming. The PLOS ONE style templates can be found at

<https://journals.plos.org/plosone/s/file?id=wjVg/PLOSOne_formatting_sample_main_body.pdf> and
<https://journals.plos.org/plosone/s/file?id=ba62/PLOSOne_formatting_sample_title_authors_affiliations.pdf>.

2. Please note that PLOS One has specific guidelines on code sharing for submissions in which author-generated code underpins the findings in the manuscript. In these cases, we expect all author-generated code to be made available without restrictions upon publication of the work. Please review our guidelines at <https://journals.plos.org/plosone/s/materials-and-software-sharing#loc-sharing-code> and ensure that your code is shared in a way that follows best practice and facilitates reproducibility and reuse.

3. Thank you for stating in your Funding Statement:

“This work was supported by Korea Environmental Industry & Technology Institute (KEITI, <https://www.keiti.re.kr/site/keiti/main.do>) through “Climate Change R&D Project for New Climate Regime,” awarded to J-HW, and funded by Korea Ministry of Environment (MOE, <https://www.mcee.go.kr/home/web/main.do>) (RS-2022-KE002096). The funder had no role in study design, data collection and analysis, decision to publish, or preparation of the manuscript”

Please provide an amended statement that declares *all* the funding or sources of support (whether external or internal to your organization) received during this study, as detailed online in our guide for authors at <http://journals.plos.org/plosone/s/submit-now>. Please also include the statement “There was no additional external funding received for this study.” in your updated Funding Statement.

Please include your amended Funding Statement within your cover letter. We will change the online submission form on your behalf.

4. We note that Figure 1 in your submission contain [map/satellite] images which may be copyrighted. All PLOS content is published under the Creative Commons Attribution License (CC BY 4.0), which means that the manuscript, images, and Supporting Information files will be freely available online, and any third party is permitted to access, download, copy, distribute, and use these materials in any way, even commercially, with proper attribution. For these reasons, we cannot publish previously copyrighted maps or satellite images created using proprietary data, such as Google software (Google Maps, Street View, and Earth). For more information, see our copyright guidelines: <http://journals.plos.org/plosone/s/licenses-and-copyright>.

We require you to either (1) present written permission from the copyright holder to publish these figures specifically under the CC BY 4.0 license, or (2) remove the figures from your submission:

a. You may seek permission from the original copyright holder of Figure 1 to publish the content specifically under the CC BY 4.0 license.

We recommend that you contact the original copyright holder with the Content Permission Form (<http://journals.plos.org/plosone/s/file?id=7c09/content-permission-form.pdf>) and the following text:
“I request permission for the open-access journal PLOS ONE to publish XXX under the Creative Commons Attribution License (CCAL) CC BY 4.0 (<http://creativecommons.org/licenses/by/4.0/>). Please be aware that this license allows unrestricted use and distribution, even commercially, by third parties. Please reply and provide explicit written permission to publish XXX under a CC BY license and complete the attached form.”

Please upload the completed Content Permission Form or other proof of granted permissions as an "Other" file with your submission.

In the figure caption of the copyrighted figure, please include the following text: “Reprinted from [ref] under a CC BY license, with permission from [name of publisher], original copyright [original copyright year].”

b. If you are unable to obtain permission from the original copyright holder to publish these figures under the CC BY 4.0 license or if the copyright holder’s requirements are incompatible with the CC BY 4.0 license, please either i) remove the figure or ii) supply a replacement figure that complies with the CC BY 4.0 license. Please check copyright information on all replacement figures and update the figure caption with source information. If applicable, please specify in the figure caption text when a figure is similar but not identical to the original image and is therefore for illustrative purposes only.
The following resources for replacing copyrighted map figures may be helpful:

USGS National Map Viewer (public domain): <http://viewer.nationalmap.gov/viewer/>
The Gateway to Astronaut Photography of Earth (public domain): <http://eol.jsc.nasa.gov/sseop/clickmap/>
Maps at the CIA (public domain): <https://www.cia.gov/library/publications/the-world-factbook/index.html> and <https://www.cia.gov/library/publications/cia-maps-publications/index.html>
NASA Earth Observatory (public domain): <http://earthobservatory.nasa.gov/>
Landsat: <http://landsat.visibleearth.nasa.gov/>
USGS EROS (Earth Resources Observatory and Science (EROS) Center) (public domain): [http://eros.usgs.gov/#](http://eros.usgs.gov/)
Natural Earth (public domain): <http://www.naturalearthdata.com/>

Answer) We thank the editorial office for raising this important issue regarding copyright and open-access licensing.

We confirm that Figure 1 does not contain any satellite imagery, screenshots, or base maps obtained from Google Maps, Google Earth, or any other proprietary mapping service. Figure 1 was created by the authors using publicly available administrative boundary data from the World Bank Official Boundaries dataset and the Administrative District Boundaries in South Korea (admdongkor) dataset.

The World Bank Official Boundaries dataset is distributed under the Creative Commons Attribution 4.0 International (CC BY 4.0) License. The admdongkor dataset is also distributed under the CC BY 4.0 License and is derived from administrative boundary data released by Statistics Korea through the Statistical Geographic Information Service (SGIS), for which attribution is required under the Korea Open Government License (KOGL) Type 1. We have preserved the required attribution to the original SGIS data source.

Accordingly, we have revised the captions of Figure 1 and all other figures containing maps to include the appropriate data sources and licensing information. We have also added the World Bank Official Boundaries dataset, the admdongkor dataset, and Statistics Korea SGIS to the References section.

Because Figure 1 and all other map figures were created by the authors using openly licensed boundary datasets with appropriate attribution and do not reproduce any copyrighted maps, satellite images, or third-party figures, no additional permission from a copyright holder is required, and a Content Permission Form is not applicable in this case.

5. If the reviewer comments include a recommendation to cite specific previously published works, please review and evaluate these publications to determine whether they are relevant and should be cited. There is no requirement to cite these works unless the editor has indicated otherwise.

[Note: HTML markup is below. Please do not edit.]

Reviewers' comments:

Reviewer's Responses to Questions

**Comments to the Author**

1. Is the manuscript technically sound, and do the data support the conclusions?

The manuscript must describe a technically sound piece of scientific research with data that supports the conclusions. Experiments must have been conducted rigorously, with appropriate controls, replication, and sample sizes. The conclusions must be drawn appropriately based on the data presented.

Reviewer #1: No

Reviewer #2: Partly

2. Has the statistical analysis been performed appropriately and rigorously?

Reviewer #1: No

Reviewer #2: Yes

3. Have the authors made all data underlying the findings in their manuscript fully available?

The [PLOS Data policy](http://track.editorialmanager.com/CL0/http:%2F%2Fwww.plosone.org%2Fstatic%2Fpolicies.action%23sharing/1/010f019e68322324-6834da5b-9fd9-4735-8b74-220838c16dba-000000/oyncjYlET6-fV6mqYtRiSgefFEMNSuZCTX9QekDgYKU=258) requires authors to make all data underlying the findings described in their manuscript fully available without restriction, with rare exception (please refer to the Data Availability Statement in the manuscript PDF file). The data should be provided as part of the manuscript or its supporting information, or deposited to a public repository. For example, in addition to summary statistics, the data points behind means, medians and variance measures should be available. If there are restrictions on publicly sharing data—e.g. participant privacy or use of data from a third party—those must be specified.

Reviewer #1: Yes

Reviewer #2: Yes

4. Is the manuscript presented in an intelligible fashion and written in standard English?

PLOS ONE does not copyedit accepted manuscripts, so the language in submitted articles must be clear, correct, and unambiguous. Any typographical or grammatical errors should be corrected at revision, so please note any specific errors here.

Reviewer #1: Yes

Reviewer #2: Yes

5. Review Comments to the Author

Please use the space provided to explain your answers to the questions above. You may also include additional comments for the author, including concerns about dual publication, research ethics, or publication ethics. (Please upload your review as an attachment if it exceeds 20,000 characters)

**Reviewer #1**

This paper aims to develop a reduced-form CMAQ model for sectoral impact analyses. The aim sounds interesting, but the paper needs significant improvement in terms of significance, method, validation, robustness, and policy insights. Below are my comments:

Answer) We thank the reviewer for the valuable comments and suggestions. We have carefully revised the manuscript and made our best efforts to address all concerns regarding the significance, methods, validation, robustness, and policy implications of this study. Detailed responses to each comment are provided below.

1. CMAQ model validation should be included. The paper uses CMAQ output as the response variable. However, it is unclear whether the CMAQ simulation is accurate or robust. Please add model validation against ground truth, such as concentrations from monitoring stations.

Answer) We thank the reviewer for highlighting the importance of evaluating the CMAQ simulations used as the response variables.

In response to this comment, we evaluated the CMAQ simulations against observations from the **AirKorea** monitoring network. CMAQ concentrations extracted at the monitoring station locations were compared with hourly ozone and PM_2.5_ observations using the correlation coefficient, root mean square error, and mean bias. We have added the validation procedure to the **Materials and methods (p. 6, lines 122–128)** and the evaluation results to the **Results** section (**pp. 11–12, lines 258–267**).

We also clarified that the objective of this study is to emulate CMAQ responses to sector- and region-specific emission changes rather than to reproduce air quality for a specific year. Accordingly, the simulations used the 2015 emission inventory together with representative 2017 meteorological fields to isolate emission-response relationships under fixed meteorological conditions. We have added a discussion of this modeling configuration and its limitations to the revised **Discussion** (**p. 22, lines 523–532**).

2. The purpose of the paper is to develop an empirical approach for identifying source impacts. The authors first calculate the emission/marginal emission impacts on concentrations from each sector-region pair, and then use ML to reproduce how each sector-region impacts others. However, I do not see the strong value of ML here. Since the authors already have a source-receptor matrix, they can directly use this matrix for policy impact analyses. So what are the policy insights gained from using ML to reproduce the source-receptor matrix? To me, the work is not really like building an RCM, but more like doing correlation analysis using the SR matrix.

Answer) We thank the reviewer for this important comment. We agree that the original manuscript did not sufficiently explain the overall framework, which may have created the impression that the proposed model was trained to reproduce a precomputed source–receptor (SR) matrix. This was not the case.

To address this issue, we substantially revised the **Materials and methods** section (**pp. 6–8, lines 131–171**) and added a new overview figure (**Fig 2**) illustrating the complete workflow. As clarified in the revised manuscript, the reduced-form models are trained directly using CMAQ simulations generated from multidimensional emission-control scenarios, rather than from a precomputed SR matrix.

Consequently, the proposed framework differs fundamentally from analyses based on SR matrices. Once trained, it can rapidly predict pollutant concentrations for arbitrary combinations of sector- and region-specific emission controls without requiring additional CMAQ simulations, enabling efficient evaluation of complex emission-control scenarios.

3. What is the spatial and temporal resolution of the emission data? Does the emission data match the CMAQ grid? This is unclear from the methods.

Answer) We thank the reviewer for this helpful comment. We agree that the description of the emission data was not sufficiently clear in the original manuscript.

The original emission inventory used in this study was the 2015 national annual emission inventory for South Korea. It was processed using SMOKE-Asia to generate CMAQ-ready emissions with the same spatial and temporal resolution as the CMAQ simulations, namely a 67 × 82 grid (9 km × 9 km) and hourly temporal resolution. Thus, the emissions used in the CMAQ simulations are fully consistent with the CMAQ grid and time steps.

We have revised the Materials and methods section to clarify the format of the original emission inventory and the SMOKE-Asia processing used to generate the CMAQ-ready emission inputs (p. 5, lines 107–110).

4. The manuscript targets PM2.5 and O3, which involve many precursors. However, the authors do not discuss the precursors in the emissions, RCM construction, or results. Adding analyses and discussions on precursor-related insights would be important. Please refer to other RCMs for more insights on precursor impacts, such as U.S. EPA’s SA-BPT and APEEP.

Answer) We thank the reviewer for this insightful comment. We agree that precursor-specific analyses can provide valuable insights into the formation mechanisms of O_3_ and PM_2.5_.

However, the objective of this study differs from that of precursor-oriented reduced-form models such as SA-BPT and APEEP. Rather than treating individual precursor species as model inputs, the proposed framework uses sector–region emission scaling factors because the primary objective is to evaluate policy-relevant sector- and region-based emission-control strategies.

Although precursor species are not explicitly represented as input variables, their effects are implicitly incorporated through the SMOKE-Asia/CMAQ modeling system. Each sector–region emission scaling factor is translated into precursor-specific emissions by SMOKE-Asia, after which CMAQ simulates atmospheric transport, chemical transformation, and deposition. Consequently, the reduced-form models learn concentration responses that already reflect precursor chemistry. As discussed in the revised manuscript, “This behavior agrees with the well-established nonlinear response of ozone to precursor emission changes in VOC-limited urban environments, where NO_x_ reductions may increase ozone concentrations” (Discussion, p. 21, lines 493–495). This demonstrates that the proposed framework preserves important precursor-related atmospheric chemistry represented by CMAQ, even though precursor species are not used directly as model inputs.

We therefore consider the proposed framework to be complementary to precursor-oriented reduced-form models. Precursor-based approaches are well suited for mechanistic analyses of individual precursor species, whereas the proposed framework focuses on rapid evaluation of policy-relevant sector- and region-based emission-control scenarios.

We have expanded the Discussion to clarify this distinction (pp. 21–22, lines 504–514). We also acknowledge that incorporating precursor-specific emission controls into the proposed framework is an important limitation of the current study and a direction for future research (p. 23, lines 539–544 and lines 549–551).

5. The ML model format is unclear. It would be helpful to add the equations of the ML models. Currently, it is unclear what the predictors and response variables are. I am also confused that the model appears to use only 119 scaling factors to predict concentrations, without involving any spatial or temporal variables. If so, how does the model generate different concentrations by grid and hour? Or are the authors generating different models for different grids and times using the same 119 predictors? If so, how to use those models for future scenario simulations? Please explain.

Answer) We thank the reviewer for this important comment. We agree that the original manuscript did not clearly describe the reduced-form modeling framework, including the predictor and response variables, or how spatial and temporal variability is represented.

To address this issue, we substantially revised the Materials and methods section (pp. 6–7, lines 131–153) by introducing explicit mathematical definitions of the predictor and response variables, the reduced-form model formulation, and the prediction procedure. We also clarified that the predictor consists of 119 sector–region emission variables (7 sectors × 17 regions; S2 Table), whereas the response is the CMAQ-simulated ozone or PM_2.5_ concentration for a specific grid cell, day, and hour.

Rather than training a single global model, the proposed framework trains an independent reduced-form model, $f_{g}^{(d,h)}$, for each grid cell *g*, day *d*, hour *h*, separately for ozone and PM_2.5_. Thus, although every model uses the same 119-dimensional predictor vector, each model represents a different spatial and temporal location.

For a new emission-control scenario, the corresponding 119-dimensional emission-control vector is provided as input to all trained reduced-form models. The resulting predictions are then combined to reconstruct the complete hourly concentration fields without additional CMAQ simulations. We have clarified this procedure in the revised Materials and methods section (p. 9, lines 188–190).

6. Robustness and sensitivity tests are needed. Currently, the results are based on one emission scenario and one approach, LASSO. I suggest adding more sensitivity tests to show that the model, approach, and results are robust.

Answer) We thank the reviewer for this valuable suggestion. We agree that robustness and sensitivity analyses are important for demonstrating the reliability of the proposed framework.

First, we would like to clarify that the proposed framework was not developed using a single emission scenario. The reduced-form models were trained using 100 emission-control scenarios generated by Latin hypercube sampling and evaluated using an independent set of 18 test scenarios. To reduce dependence on a particular train–test partition, the random train–test split was repeated 10 times, and all reported results were averaged over the repeated experiments.

In response to the reviewer’s comment, we expanded the comparison of machine learning methods by evaluating LASSO, SVR-RBF, and XGBoost. LASSO achieved the highest prediction accuracy while also requiring the shortest training time, making it the most suitable method for constructing more than 16 million reduced-form models.

We also conducted a sensitivity analysis of the response-variable transformation by comparing the untransformed, logarithmic, logit, and adaptive logit formulations. Although all four transformations produced similar mean prediction errors, the proposed adaptive logit transformation substantially reduced the maximum RMSE, indicating improved robustness against localized extreme prediction errors.

The revised manuscript has been updated accordingly. The comparison of machine learning methods has been incorporated into the Materials and methods (p. 7, lines 154–164) and Results (pp. 12–13, lines 270–289) sections. The response-variable transformation analysis has been incorporated into the Materials and methods (p. 8, lines 172–187) and Results (p. 13, lines 291–308) sections.

7. It is unclear how the authors derived the sectoral influence. Have the authors compared the results with sectoral impacts directly from CMAQ? How do they define the most influential variables? Are the results robust?

Answer) We thank the reviewer for this important comment. We would like to clarify that the proposed influence measures were not derived from CMAQ source-apportionment or sensitivity analysis tools. Rather, they were inferred from the fitted LASSO-based reduced-form models trained using CMAQ simulation outputs. Because all predictor variables are dimensionless emission scaling factors defined on a common scale, the fitted coefficients can be interpreted as coefficient-based sensitivities within the surrogate modeling framework.

To address the reviewer’s concern, we substantially revised the Materials and methods section by providing explicit mathematical definitions of both the nationwide sectoral influence analysis and the regional sector–region influence analysis and added two new schematic figures illustrating the calculation procedures (Fig 3 and S1 Fig; pp. 9–11, lines 192–245). We also clarified that the five sector–region variables with the largest influence ratios were defined as the most influential variables for each region.

To evaluate robustness, we recalculated the sectoral influence ratios for each of the 10 repeated random train–test splits. The standard deviations ranged from only 0.1 to 0.2 percentage points, indicating that the estimated influence patterns were highly stable. Because these values were very small, they are reported in the Results section rather than shown graphically (p. 16, lines 375–378).

Finally, we expanded the Discussion to clarify the interpretation of the proposed influence measures (p. 20, lines 468–473). Specifically, we emphasize that the reported influence ratios should be interpreted within the sampled scenario space rather than as the absolute real-world importance of the corresponding emission sectors.

8. The model uses the log-transformed response variable. Have the authors done sensitivity tests using the linear format? Also, if the purpose is to solve nonlinearity, why not use tree-based approaches?

Answer) We thank the reviewer for this helpful comment. As described in our response to Comment 6, we conducted a sensitivity analysis comparing the untransformed, logarithmic, logit, and adaptive logit response-variable transformations. We also compared LASSO, SVR-RBF, and XGBoost, where XGBoost represents a tree-based approach. LASSO achieved the highest prediction accuracy while also requiring the shortest training time, making it the most suitable method for constructing more than 16 million reduced-form models.

The corresponding comparisons have been added to the revised Materials and methods (p. 7, lines 154–164; p. 8, lines 172–187) and Results (pp. 12–13, lines 270–308) sections.

9. Line 156: It is unclear how “little variation” is defined. What is the criterion? If the authors only want to remove ocean cells, that is probably fine. But please be consistent and explicitly list the criteria to avoid potential biases.

Answer) We thank the reviewer for pointing out the need to clearly define the criterion for “little variation.” We agree that explicitly stating the criterion helps avoid ambiguity and potential bias.

In this study, “little variation” was defined as cases where the response variable contained five or fewer unique values in the training data. In such cases, LASSO model training was replaced by a constant predictor equal to the mean of the training responses because reliable model fitting was not possible. This criterion was applied uniformly to all grid cells, days, and hours throughout the simulation domain and was not intended to identify or remove ocean cells or any other specific geographic regions.

We revised the Materials and methods section to explicitly describe this criterion and its rationale (p. 7, lines 156–162). We also added a quantitative evaluation in the Results section and S5 Table, showing that such cases occurred primarily outside the 17 administrative regions and were extremely rare within the primary study area (p. 14, lines 311–320).

10. Lines 188–194: Please explain the meaning of the coefficients from the LASSO models and the meaning of the influence ratio. To my understanding, the predictors are emission scaling ratios. Therefore, it is unclear what it means to sum up the coefficients for scaling factors.

Answer) We thank the reviewer for this valuable comment. As explained in our response to Comment 7, the fitted LASSO coefficients should be interpreted as coefficient-based sensitivities within the surrogate modeling framework rather than as physical source contributions from CMAQ. Because all predictor variables are dimensionless emission scaling factors defined on a common scale, their coefficients can be directly compared and aggregated.

To address the reviewer’s concern, we substantially revised the Materials and methods section by providing explicit mathematical definitions of the influence ratios and added schematic figures illustrating the calculation procedures (Fig 3 and S1 Fig; pp. 9–11, lines 192–245). We also expanded the Discussion to clarify the interpretation of the proposed influence measures (p. 20, lines 468–473).

11. Line 235: This part should be moved to the Methods section.

Answer) We thank the reviewer for this helpful suggestion. As suggested, we moved this description from the Results section to the Materials and methods section (p. 11, lines 247–255) of the revised manuscript..

**Reviewer #2**

This study developed a fast reduced-form air quality model using LASSO regressions of 119 CMAQ simulations to efficiently predict ozone and PM2.5 responses to emission changes across South Korea. It’s great that the authors also develop a user-friendly Web server tool. I think this study provides an useful tool, although I think it lacks technical details and deeper scientific insights. I have the following suggestions

Answer) We thank the reviewer for the encouraging comments and constructive suggestions. We appreciate the recognition of the utility of the proposed framework and web-based tool. In response, we have revised the manuscript to provide additional technical details and strengthen the scientific discussion. Detailed responses to each comment are provided below.

Method

1. How long is your spin-up time for CMAQ simulations?

Answer) We thank the reviewer for this question. A 7-day spin-up period was applied to all CMAQ simulations. This information has been added to the “CMAQ simulations” subsection of the revised Materials and methods (p. 6, lines 116–118).

2. Developing independent model for every single hour and grid cell sounds a lot. Are there previous studies that used a similar approach? How many data points were used to train each model?

Answer) We thank the reviewer for this important comment.

Each reduced-form model was trained using 100 emission-control scenarios, with the remaining 18 scenarios reserved for independent testing. Each training sample consisted of the 119 sector–region emission scaling factors as predictors and the CMAQ-simulated pollutant concentration for a specific grid cell, day, and hour as the response. We have clarified this in the revised Materials and methods (p. 8, lines 165–171).

The use of independent response functions for individual grid cells or time periods is consistent with previous reduced-form air quality modeling studies. In the revised manuscript, we have added relevant references and clarified that the proposed framework extends these approaches by developing independent grid–day–hour-specific reduced-form models to emulate hourly CMAQ concentration responses (p. 7, lines 149–153).

3. What are the input emission variables? Please provide a list.

Answer) We thank the reviewer for this helpful comment. The input variables of the reduced-form models are the 119 sector–region emission variables, corresponding to all combinations of the seven emission sectors (Power, Industry, Residential, Solvent, Mobile, Agriculture, and Others) and the 17 provincial-level administrative regions in South Korea. To address the reviewer’s comment, we have added S2 Table, which lists the 17 regions and the seven emission sectors that define the 119 sector–region emission variables, and revised the Materials and methods section accordingly (p. 5, lines 101–102).

4. It seems that the underlying assumption is that all the emission variables would increase or decrease by the same ratio. If so, it’s worth clarifying this as a limitation because some emission control strategies may reduce only emissions of certain pollutants.

Answer) We thank the reviewer for this important comment. The reviewer’s understanding is correct. In the proposed framework, each input variable is a sector–region emission scaling factor. Consequently, all emissions within a given sector–region are scaled by the same factor when generating an emission-control scenario.

This modeling choice was made because the objective of this study is to evaluate policy-relevant sector- and region-based emission-control strategies, for which emission controls are typically implemented at the sectoral and regional levels rather than for individual pollutant species.

We agree that this assumption represents an important limitation of the current framework. Specifically, the proposed framework cannot directly represent emission-control strategies in which different pollutant species within the same sector–region are controlled independently. We have clarified this assumption in the Materials and methods section (p. 5, lines 103–105) and added it as a limitation in the Discussion (p. 23, lines 539–544) section.

Results

5. Interpretation of LASSO coefficients

5.1 “23.5 variables for ozone” - why would there be 0.5 variable? Or did you average the number of variables for grid cells? I think it’s also worth reporting how many distinct variables across models.

Answer) We thank the reviewer for this helpful comment. The value “23.5 variables” refers to the average number of input variables with nonzero coefficients per reduced-form model, averaged across all grid cells, days, hours, and repeated random train–test splits. Thus, each individual reduced-form model selected an integer number of variables, whereas the reported value represents the average across all fitted models.

To make this clearer, we have revised the Results section to explicitly explain the meaning of this value (p. 16, lines 362–372). We have also added S3 Fig, which shows the distribution of the numbers of selected variables across all fitted reduced-form models, providing a more complete view of the sparsity of the fitted LASSO models.

5.2 Could you also report what these important variables are? Are there correlations between these variables?

Answer) We thank the reviewer for this helpful suggestion. Because the reported value of 23.5 variables represents the average number of variables selected per reduced-form model, there is no single set of 23.5 variables shared across all models. The selected variables vary according to the grid cell, day, and hour represented by each reduced-form model.

Instead, we summarized the selected variables through nationwide sectoral influence and regional sector–region influence analyses. The regional analysis showed that, for ozone, the Mobile sector was consistently represented among the five most influential sector–region variables across all regions, whereas for PM_2.5_, the influential variables varied more among regions, with the Agriculture, Industry, Others, and Mobile sectors most frequently represented. The revised Results section also highlights that influential variables often originated from neighboring regions, reflecting the regional transport effects represented in the CMAQ simulations (pp. 17–18, lines 408–413).

6. It’s not clear to me how influence ratios across regions were calculated

Answer) We thank the reviewer for this helpful comment. We agree that the procedure for calculating the regional influence ratios was not sufficiently clear in the original manuscript.

To address this issue, we have substantially revised the Materials and methods section (pp. 9–11, lines 192–245) by providing explicit mathematical definitions of both the nationwide sectoral influence analysis and the regional sector–region influence analysis. We have also added two schematic figures illustrating the complete calculation procedures: Fig 3 for the nationwide sectoral influence analysis and S1 Fig for the regional sector–region influence analysis.

In brief, the regional influence ratio was calculated by aggregating the absolute values of the LASSO coefficients associated with each sector–region emission variable across all reduced-form models corresponding to the grid cells within the receptor region and then normalizing by the total absolute coefficient sum over all 119 sector–region emission variables. The influence direction was determined separately from the aggregated signed coefficients.

7. Figure resolution is low

Answer) We thank the reviewer for this comment.

We have carefully reviewed and revised all figures in the revised manuscript and Supporting information to improve their readability and ensure that they meet the PLOS ONE figure requirements. In particular, the axes, labels, legends, and other graphical elements have been refined to improve clarity. We also verified all figures using the PLOS NAAS figure assessment tool, and they satisfy the journal's technical specifications.

The revised figures have been incorporated into the revised manuscript and Supporting information.

8. Line 239-242: could you explain why reductions in variables lead to increase in concentrations? And are you discussing the influence on average concentrations across grid cells and the four months?

Answer) We thank the reviewer for this helpful comment.

The influential sector–region variables for ozone in Seoul were predominantly associated with negative aggregated LASSO coefficient sums. This indicates that increasing the corresponding emission scaling factors is associated with lower predicted ozone concentrations, whereas reducing them is associated with higher ozone concentrations within the proposed framework. This behavior is consistent with the well-established nonlinear chemistry of ozone formation in VOC-limited urban environments, where reductions in NO_x_ emissions may increase ozone concentrations because of reduced NO titration and nonlinear precursor interactions. We have added this explanation, together with the relevant references, to the revised Discussion (p. 21, lines 493–495) section.

To further support this interpretation, we examined the corresponding CMAQ simulations used for model development. We found that scenarios with substantially increased Mobile sector emissions in Seoul and Gyeonggi also produced lower annual mean CMAQ-simulated ozone concentrations in these regions, consistent with the direction indicated by the aggregated LASSO coefficients. This result has also been added to the revised Discussion (p. 21, lines 489–493) section.

The reported concentration changes are spatial and temporal averages rather than values for individual grid cells or time steps. Specifically, they were calculated by averaging the predicted concentrations over all grid cells within the target administrative region and over all hours of the four representative months (123 days in total). We have clarified this in the revised manuscript (p. 18, lines 424–426).

6. PLOS authors have the option to publish the peer review history of their article (what does this mean?). If published, this will include your full peer review and any attached files.

If you choose “no”, your identity will remain anonymous but your review may still be made public.


**Do you want your identity to be public for this peer review?** For information about this choice, including consent withdrawal, please see our [Privacy Policy](https://track.editorialmanager.com/CL0/https:%2F%2Fwww.plos.org%2Fprivacy-policy/1/010f019e68322324-6834da5b-9fd9-4735-8b74-220838c16dba-000000/6qE-GBmZenlsNgH3msi_T06ipiFH2jUOgPJOnw4ut_k=258).

Reviewer #1: **Yes:** Yuzhou Wang

Reviewer #2: No

[NOTE: If reviewer comments were submitted as an attachment file, they will be attached to this email and accessible via the submission site. Please log into your account, locate the manuscript record, and check for the action link "View Attachments". If this link does not appear, there are no attachment files.]

To ensure your figures meet our technical requirements, please review our figure guidelines: <https://journals.plos.org/plosone/s/figures>

You may also use PLOS’s free figure tool, NAAS, to help you prepare publication quality figures: <https://journals.plos.org/plosone/s/figures#loc-tools-for-figure-preparation>.

NAAS will assess whether your figures meet our technical requirements by comparing each figure against our figure specifications.

*In compliance with data protection regulations, you may request that we remove your personal registration details at any time.* [*(Remove my information/details)*](https://track.editorialmanager.com/CL0/https:%2F%2Fwww.editorialmanager.com%2Fpone%2Flogin.asp%3Fa=r/1/010f019e68322324-6834da5b-9fd9-4735-8b74-220838c16dba-000000/_IwDJgfqFj2wEemdpurebYFa9HgACPFkFgcy0QAACck=258)*. Please contact the publication office if you have any questions.*
