# Supplementary material for: A LASSO-based reduced-form CMAQ model for predicting ozone and PM2.5 responses to emission changes in South Korea
Source: PLoS One. 2026 Sep 15;21(9):e0347073. doi: 10.1371/journal.pone.0347073 (PMC13577401; doi:10.1371/journal.pone.0347073)
Supplement: Peer review history

# Peer review: Decision Letter 0

**DOI:** 10.1371/journal.pone.0347073.r001

**Authors:** Yangyang Xu

27 May 2026

Dear Dr. Hwang,

Thank you for submitting your manuscript to PLOS ONE. After careful consideration, we feel that it has merit but does not fully meet PLOS ONE’s publication criteria as it currently stands. Therefore, we invite you to submit a revised version of the manuscript that addresses the points raised during the review process.

Please submit your revised manuscript by Jul 11 2026 11:59PM. If you will need more time than this to complete your revisions, please reply to this message or contact the journal office at plosone@plos.org. When you're ready to submit your revision, log on to https://www.editorialmanager.com/pone/ and select the 'Submissions Needing Revision' folder to locate your manuscript file.

- A letter that responds to each point raised by the academic editor and reviewer(s). You should upload this letter as a separate file labeled 'Response to Reviewers'.
- A marked-up copy of your manuscript that highlights changes made to the original version. You should upload this as a separate file labeled 'Revised Manuscript with Track Changes'.
- An unmarked version of your revised paper without tracked changes. You should upload this as a separate file labeled 'Manuscript'.

If you would like to make changes to your financial disclosure, please include your updated statement in your cover letter. Guidelines for resubmitting your figure files are available below the reviewer comments at the end of this letter.

If applicable, we recommend that you deposit your laboratory protocols in protocols.io to enhance the reproducibility of your results. Protocols.io assigns your protocol its own identifier (DOI) so that it can be cited independently in the future. For instructions see: https://journals.plos.org/plosone/s/submission-guidelines#loc-laboratory-protocols. Additionally, PLOS ONE offers an option for publishing peer-reviewed Lab Protocol articles, which describe protocols hosted on protocols.io. Read more information on sharing protocols at https://plos.org/protocols?utm_medium=editorial-email&utm_source=authorletters&utm_campaign=protocols.

As the corresponding author, your ORCID iD is verified in the submission system and will appear in the published article. PLOS supports the use of ORCID, and we encourage all coauthors to register for an ORCID iD and use it as well. Please encourage your coauthors to verify their ORCID iD within the submission system before final acceptance, as unverified ORCID iDs will not appear in the published article. *Only*  the individual author can complete the verification step; PLOS staff *cannot*  verify ORCID iDs on behalf of authors.

We look forward to receiving your revised manuscript.

Kind regards,

Yangyang Xu

Academic Editor

PLOS One

Journal Requirements:

When submitting your revision, we need you to address these additional requirements.

1. Please ensure that your manuscript meets PLOS ONE's style requirements, including those for file naming. The PLOS ONE style templates can be found at

https://journals.plos.org/plosone/s/file?id=wjVg/PLOSOne_formatting_sample_main_body.pdf and

https://journals.plos.org/plosone/s/file?id=ba62/PLOSOne_formatting_sample_title_authors_affiliations.pdf.

2. Please note that PLOS One has specific guidelines on code sharing for submissions in which author-generated code underpins the findings in the manuscript. In these cases, we expect all author-generated code to be made available without restrictions upon publication of the work. Please review our guidelines at https://journals.plos.org/plosone/s/materials-and-software-sharing#loc-sharing-code and ensure that your code is shared in a way that follows best practice and facilitates reproducibility and reuse.

3. Thank you for stating in your Funding Statement:

“This work was supported by Korea Environmental Industry & Technology Institute (KEITI, https://www.keiti.re.kr/site/keiti/main.do) through “Climate Change R&D Project for New Climate Regime,” awarded to J-HW, and funded by Korea Ministry of Environment (MOE, https://www.mcee.go.kr/home/web/main.do) (RS-2022-KE002096). The funder had no role in study design, data collection and analysis, decision to publish, or preparation of the manuscript”

Please provide an amended statement that declares *all* the funding or sources of support (whether external or internal to your organization) received during this study, as detailed online in our guide for authors at http://journals.plos.org/plosone/s/submit-now.  Please also include the statement “There was no additional external funding received for this study.” in your updated Funding Statement.

Please include your amended Funding Statement within your cover letter. We will change the online submission form on your behalf.

4. We note that Figure 1 in your submission contain [map/satellite] images which may be copyrighted. All PLOS content is published under the Creative Commons Attribution License (CC BY 4.0), which means that the manuscript, images, and Supporting Information files will be freely available online, and any third party is permitted to access, download, copy, distribute, and use these materials in any way, even commercially, with proper attribution. For these reasons, we cannot publish previously copyrighted maps or satellite images created using proprietary data, such as Google software (Google Maps, Street View, and Earth). For more information, see our copyright guidelines: http://journals.plos.org/plosone/s/licenses-and-copyright.

We require you to either (1) present written permission from the copyright holder to publish these figures specifically under the CC BY 4.0 license, or (2) remove the figures from your submission:

a. You may seek permission from the original copyright holder of Figure 1 to publish the content specifically under the CC BY 4.0 license.

We recommend that you contact the original copyright holder with the Content Permission Form (http://journals.plos.org/plosone/s/file?id=7c09/content-permission-form.pdf) and the following text:

“I request permission for the open-access journal PLOS ONE to publish XXX under the Creative Commons Attribution License (CCAL) CC BY 4.0 (http://creativecommons.org/licenses/by/4.0/). Please be aware that this license allows unrestricted use and distribution, even commercially, by third parties. Please reply and provide explicit written permission to publish XXX under a CC BY license and complete the attached form.”

Please upload the completed Content Permission Form or other proof of granted permissions as an "Other" file with your submission.

In the figure caption of the copyrighted figure, please include the following text: “Reprinted from [ref] under a CC BY license, with permission from [name of publisher], original copyright [original copyright year].”

b. If you are unable to obtain permission from the original copyright holder to publish these figures under the CC BY 4.0 license or if the copyright holder’s requirements are incompatible with the CC BY 4.0 license, please either i) remove the figure or ii) supply a replacement figure that complies with the CC BY 4.0 license. Please check copyright information on all replacement figures and update the figure caption with source information. If applicable, please specify in the figure caption text when a figure is similar but not identical to the original image and is therefore for illustrative purposes only.

The following resources for replacing copyrighted map figures may be helpful:

USGS National Map Viewer (public domain): http://viewer.nationalmap.gov/viewer/

The Gateway to Astronaut Photography of Earth (public domain): http://eol.jsc.nasa.gov/sseop/clickmap/

Maps at the CIA (public domain): https://www.cia.gov/library/publications/the-world-factbook/index.html and https://www.cia.gov/library/publications/cia-maps-publications/index.html

NASA Earth Observatory (public domain): http://earthobservatory.nasa.gov/

Landsat: http://landsat.visibleearth.nasa.gov/

USGS EROS (Earth Resources Observatory and Science (EROS) Center) (public domain): http://eros.usgs.gov/#

Natural Earth (public domain): http://www.naturalearthdata.com/

5. If the reviewer comments include a recommendation to cite specific previously published works, please review and evaluate these publications to determine whether they are relevant and should be cited. There is no requirement to cite these works unless the editor has indicated otherwise.

[Note: HTML markup is below. Please do not edit.]

Reviewers' comments:

Reviewer's Responses to Questions

**Comments to the Author**

1. Is the manuscript technically sound, and do the data support the conclusions?

Reviewer #1: No

Reviewer #2: Partly

**********

2. Has the statistical analysis been performed appropriately and rigorously?

Reviewer #1: No

Reviewer #2: Yes

**********

3. Have the authors made all data underlying the findings in their manuscript fully available?

The PLOS Data policy

Reviewer #1: Yes

Reviewer #2: Yes

**********

4. Is the manuscript presented in an intelligible fashion and written in standard English?

Reviewer #1: Yes

Reviewer #2: Yes

**********

Reviewer #1: This paper aims to develop a reduced-form CMAQ model for sectoral impact analyses. The aim sounds interesting, but the paper needs significant improvement in terms of significance, method, validation, robustness, and policy insights. Below are my comments:

1. CMAQ model validation should be included. The paper uses CMAQ output as the response variable. However, it is unclear whether the CMAQ simulation is accurate or robust. Please add model validation against ground truth, such as concentrations from monitoring stations.

2. The purpose of the paper is to develop an empirical approach for identifying source impacts. The authors first calculate the emission/marginal emission impacts on concentrations from each sector-region pair, and then use ML to reproduce how each sector-region impacts others. However, I do not see the strong value of ML here. Since the authors already have a source-receptor matrix, they can directly use this matrix for policy impact analyses. So what are the policy insights gained from using ML to reproduce the source-receptor matrix? To me, the work is not really like building an RCM, but more like doing correlation analysis using the SR matrix.

3. What is the spatial and temporal resolution of the emission data? Does the emission data match the CMAQ grid? This is unclear from the methods.

4. The manuscript targets PM2.5 and O3, which involve many precursors. However, the authors do not discuss the precursors in the emissions, RCM construction, or results. Adding analyses and discussions on precursor-related insights would be important. Please refer to other RCMs for more insights on precursor impacts, such as U.S. EPA’s SA-BPT and APEEP.

5. The ML model format is unclear. It would be helpful to add the equations of the ML models. Currently, it is unclear what the predictors and response variables are. I am also confused that the model appears to use only 119 scaling factors to predict concentrations, without involving any spatial or temporal variables. If so, how does the model generate different concentrations by grid and hour? Or are the authors generating different models for different grids and times using the same 119 predictors? If so, how to use those models for future scenario simulations? Please explain.

6. Robustness and sensitivity tests are needed. Currently, the results are based on one emission scenario and one approach, LASSO. I suggest adding more sensitivity tests to show that the model, approach, and results are robust.

7. It is unclear how the authors derived the sectoral influence. Have the authors compared the results with sectoral impacts directly from CMAQ? How do they define the most influential variables? Are the results robust?

8. The model uses the log-transformed response variable. Have the authors done sensitivity tests using the linear format? Also, if the purpose is to solve nonlinearity, why not use tree-based approaches?

9. Line 156: It is unclear how “little variation” is defined. What is the criterion? If the authors only want to remove ocean cells, that is probably fine. But please be consistent and explicitly list the criteria to avoid potential biases.

10. Lines 188–194: Please explain the meaning of the coefficients from the LASSO models and the meaning of the influence ratio. To my understanding, the predictors are emission scaling ratios. Therefore, it is unclear what it means to sum up the coefficients for scaling factors.

11. Line 235: This part should be moved to the Methods section.

Reviewer #2: This study developed a fast reduced-form air quality model using LASSO regressions of 119 CMAQ simulations to efficiently predict ozone and PM2.5 responses to emission changes across South Korea. It’s great that the authors also develop a user-friendly Web server tool. I think this study provides an useful tool, although I think it lacks technical details and deeper scientific insights. I have the following suggestions

Method

- How long is your spin-up time for CMAQ simulations?

- Developing independent model for every single hour and grid cell sounds a lot. Are there previous studies that used a similar approach? How many data points were used to train each model?

- What are the input emission variables? Please provide a list.

- It seems that the underlying assumption is that all the emission variables would increase or decrease by the same ratio. If so, it’s worth clarifying this as a limitation because some emission control strategies may reduce only emissions of certain pollutants.

Results

- Interpretation of LASSO coefficients

o “23.5 variables for ozone” - why would there be 0.5 variable? Or did you average the number of variables for grid cells? I think it’s also worth reporting how many distinct variables across models.

o Could you also report what these important variables are? Are there correlations between these variables?

- It’s not clear to me how influence ratios across regions were calculated

- Figure resolution is low

- Line 239-242: could you explain why reductions in variables lead to increase in concentrations? And are you discussing the influence on average concentrations across grid cells and the four months?

**********

what does this mean?). If published, this will include your full peer review and any attached files.

If you choose “no”, your identity will remain anonymous but your review may still be made public.

**Do you want your identity to be public for this peer review?**  For information about this choice, including consent withdrawal, please see our Privacy Policy

Reviewer #1: **Yes:** Yuzhou Wang

Reviewer #2: No

**********

[NOTE: If reviewer comments were submitted as an attachment file, they will be attached to this email and accessible via the submission site. Please log into your account, locate the manuscript record, and check for the action link "View Attachments". If this link does not appear, there are no attachment files.]

To ensure your figures meet our technical requirements, please review our figure guidelines: https://journals.plos.org/plosone/s/figures

You may also use PLOS’s free figure tool, NAAS, to help you prepare publication quality figures: https://journals.plos.org/plosone/s/figures#loc-tools-for-figure-preparation.

NAAS will assess whether your figures meet our technical requirements by comparing each figure against our figure specifications.

# Author comment: Author response to Decision Letter 1

**DOI:** 10.1371/journal.pone.0347073.r002

23 Jul 2026

Reviewer #1

This paper aims to develop a reduced-form CMAQ model for sectoral impact analyses. The aim sounds interesting, but the paper needs significant improvement in terms of significance, method, validation, robustness, and policy insights. Below are my comments:

Answer) We thank the reviewer for the valuable comments and suggestions. We have carefully revised the manuscript and made our best efforts to address all concerns regarding the significance, methods, validation, robustness, and policy implications of this study. Detailed responses to each comment are provided below.

1. CMAQ model validation should be included. The paper uses CMAQ output as the response variable. However, it is unclear whether the CMAQ simulation is accurate or robust. Please add model validation against ground truth, such as concentrations from monitoring stations.

Answer) We thank the reviewer for highlighting the importance of evaluating the CMAQ simulations used as the response variables.

In response to this comment, we evaluated the CMAQ simulations against observations from the AirKorea monitoring network. CMAQ concentrations extracted at the monitoring station locations were compared with hourly ozone and PM2.5 observations using the correlation coefficient, root mean square error, and mean bias. We have added the validation procedure to the Materials and methods (p. 6, lines 122–128) and the evaluation results to the Results section (pp. 11–12, lines 258–267).

We also clarified that the objective of this study is to emulate CMAQ responses to sector- and region-specific emission changes rather than to reproduce air quality for a specific year. Accordingly, the simulations used the 2015 emission inventory together with representative 2017 meteorological fields to isolate emission-response relationships under fixed meteorological conditions. We have added a discussion of this modeling configuration and its limitations to the revised Discussion (p. 22, lines 523–532).

2. The purpose of the paper is to develop an empirical approach for identifying source impacts. The authors first calculate the emission/marginal emission impacts on concentrations from each sector-region pair, and then use ML to reproduce how each sector-region impacts others. However, I do not see the strong value of ML here. Since the authors already have a source-receptor matrix, they can directly use this matrix for policy impact analyses. So what are the policy insights gained from using ML to reproduce the source-receptor matrix? To me, the work is not really like building an RCM, but more like doing correlation analysis using the SR matrix.

Answer) We thank the reviewer for this important comment. We agree that the original manuscript did not sufficiently explain the overall framework, which may have created the impression that the proposed model was trained to reproduce a precomputed source–receptor (SR) matrix. This was not the case.

To address this issue, we substantially revised the Materials and methods section (pp. 6–8, lines 131–171) and added a new overview figure (Fig 2) illustrating the complete workflow. As clarified in the revised manuscript, the reduced-form models are trained directly using CMAQ simulations generated from multidimensional emission-control scenarios, rather than from a precomputed SR matrix.

Consequently, the proposed framework differs fundamentally from analyses based on SR matrices. Once trained, it can rapidly predict pollutant concentrations for arbitrary combinations of sector- and region-specific emission controls without requiring additional CMAQ simulations, enabling efficient evaluation of complex emission-control scenarios.

3. What is the spatial and temporal resolution of the emission data? Does the emission data match the CMAQ grid? This is unclear from the methods.

Answer) We thank the reviewer for this helpful comment. We agree that the description of the emission data was not sufficiently clear in the original manuscript.

The original emission inventory used in this study was the 2015 national annual emission inventory for South Korea. It was processed using SMOKE-Asia to generate CMAQ-ready emissions with the same spatial and temporal resolution as the CMAQ simulations, namely a 67 × 82 grid (9 km × 9 km) and hourly temporal resolution. Thus, the emissions used in the CMAQ simulations are fully consistent with the CMAQ grid and time steps.

We have revised the Materials and methods section to clarify the format of the original emission inventory and the SMOKE-Asia processing used to generate the CMAQ-ready emission inputs (p. 5, lines 107–110).

4. The manuscript targets PM2.5 and O3, which involve many precursors. However, the authors do not discuss the precursors in the emissions, RCM construction, or results. Adding analyses and discussions on precursor-related insights would be important. Please refer to other RCMs for more insights on precursor impacts, such as U.S. EPA’s SA-BPT and APEEP.

Answer) We thank the reviewer for this insightful comment. We agree that precursor-specific analyses can provide valuable insights into the formation mechanisms of O3 and PM2.5.

However, the objective of this study differs from that of precursor-oriented reduced-form models such as SA-BPT and APEEP. Rather than treating individual precursor species as model inputs, the proposed framework uses sector–region emission scaling factors because the primary objective is to evaluate policy-relevant sector- and region-based emission-control strategies.

Although precursor species are not explicitly represented as input variables, their effects are implicitly incorporated through the SMOKE-Asia/CMAQ modeling system. Each sector–region emission scaling factor is translated into precursor-specific emissions by SMOKE-Asia, after which CMAQ simulates atmospheric transport, chemical transformation, and deposition. Consequently, the reduced-form models learn concentration responses that already reflect precursor chemistry. As discussed in the revised manuscript, “This behavior agrees with the well-established nonlinear response of ozone to precursor emission changes in VOC-limited urban environments, where NOx reductions may increase ozone concentrations” (Discussion, p. 21, lines 493–495). This demonstrates that the proposed framework preserves important precursor-related atmospheric chemistry represented by CMAQ, even though precursor species are not used directly as model inputs.

We therefore consider the proposed framework to be complementary to precursor-oriented reduced-form models. Precursor-based approaches are well suited for mechanistic analyses of individual precursor species, whereas the proposed framework focuses on rapid evaluation of policy-relevant sector- and region-based emission-control scenarios.

We have expanded the Discussion to clarify this distinction (pp. 21–22, lines 504–514). We also acknowledge that incorporating precursor-specific emission controls into the proposed framework is an important limitation of the current study and a direction for future research (p. 23, lines 539–544 and lines 549–551).

5. The ML model format is unclear. It would be helpful to add the equations of the ML models. Currently, it is unclear what the predictors and response variables are. I am also confused that the model appears to use only 119 scaling factors to predict concentrations, without involving any spatial or temporal variables. If so, how does the model generate different concentrations by grid and hour? Or are the authors generating different models for different grids and times using the same 119 predictors? If so, how to use those models for future scenario simulations? Please explain.

Answer) We thank the reviewer for this important comment. We agree that the original manuscript did not clearly describe the reduced-form modeling framework, including the predictor and response variables, or how spatial and temporal variability is represented.

To address this issue, we substantially revised the Materials and methods section (pp. 6–7, lines 131–153) by introducing explicit mathematical definitions of the predictor and response variables, the reduced-form model formulation, and the prediction procedure. We also clarified that the predictor consists of 119 sector–region emission variables (7 sectors × 17 regions; S2 Table), whereas the response is the CMAQ-simulated ozone or PM2.5 concentration for a specific grid cell, day, and hour.

Rather than training a single global model, the proposed framework trains an independent reduced-form model, f_g^((d,h)), for each grid cell g, day d, hour h, separately for ozone and PM2.5. Thus, although every model uses the same 119-dimensional predictor vector, each model represents a different spatial and temporal location.

For a new emission-control scenario, the corresponding 119-dimensional emission-control vector is provided as input to all trained reduced-form models. The resulting predictions are then combined to reconstruct the complete hourly concentration fields without additional CMAQ simulations. We have clarified this procedure in the revised Materials and methods section (p. 9, lines 188–190).

6. Robustness and sensitivity tests are needed. Currently, the results are based on one emission scenario and one approach, LASSO. I suggest adding more sensitivity tests to show that the model, approach, and results are robust.

Answer) We thank the reviewer for this valuable suggestion. We agree that robustness and sensitivity analyses are important for demonstrating the reliability of the proposed framework.

First, we would like to clarify that the proposed framework was not developed using a single emission scenario. The reduced-form models were trained using 100 emission-control scenarios generated by Latin hypercube sampling and evaluated using an independent set of 18 test scenarios. To reduce dependence on a particular train–test partition, the random train–test split was repeated 10 times, and all reported results were averaged over the repeated experiments.

In response to the reviewer’s comment, we expanded the comparison of machine learning methods by evaluating LASSO, SVR-RBF, and XGBoost. LASSO achieved the highest prediction accuracy while also requiring the shortest training time, making it the most suitable method for constructing more than 16 million reduced-form models.

We also conducted a sensitivity analysis of the response-variable transformation by comparing the untransformed, logarithmic, logit, and adaptive logit formulations. Although all four transformations produced similar mean prediction errors, the proposed adaptive logit transformation substantially reduced the maximum RMSE, indicating improved robustness against localized extreme prediction errors.

The revised manuscript has been updated accordingly. The comparison of machine learning methods has been incorporated into the Materials and methods (p. 7, lines 154–164) and Results (pp. 12–13, lines 270–289) sections. The response-variable transformation analysis has been incorporated into the Materials and methods (p. 8, lines 172–187) and Results (p. 13, lines 291–308) sections.

7. It is unclear how the authors derived the sectoral influence. Have the authors compared the results with sectoral impacts directly from CMAQ? How do they define the most influential variables? Are the results robust?

Answer) We thank the reviewer for this important comment. We would like to clarify that the proposed influence measures were not derived from CMAQ source-apportionment or sensitivity analysis tools. Rather, they were inferred from the fitted LASSO-based reduced-form models trained using CMAQ simulation outputs. Because all predictor variables are dimensionless emission scaling factors defined on a common scale, the fitted coefficients can be interpreted as coefficient-based sensitivities within the surrogate modeling framework.

To address the reviewer’s concern, we substantially revised the Materials and methods section by providing explicit mathematical definitions of both the nationwide sectoral influence analysis and the regional sector–region influence analysis and added two new schematic figures illustrating the calculation procedures (Fig 3 and S1 Fig; pp. 9–11, lines 192–245). We also clarified that the five sector–region variables with the largest influence ratios were defined as the most influential variables for each region.

To evaluate robustness, we recalculated the sectoral influence ratios for each of the 10 repeated random train–test splits. The standard deviations ranged from only 0.1 to 0.2 percentage points, indicating that the estimated influence patterns were highly stable. Because these values were very small, they are reported in the Results section rather than shown graphically (p. 16, lines 375–378).

Finally, we expanded the Discussion to clarify the interpretation of the proposed influence measures (p. 20, lines 468–473). Specifically, we emphasize that the reported influence ratios should be interpreted within the sampled scenario space rather than as the absolute real-world importance of the corresponding emission sectors.

8. The model uses the log-transformed response variable. Have the authors done sensitivity tests using the linear format? Also, if the purpose is to solve nonlinearity, why not use tree-based approaches?

Answer) We thank the reviewer for this helpful comment. As described in our response to Comment 6, we conducted a sensitivity analysis comparing the untransformed, logarithmic, logit, and adaptive logit response-variable transformations. We also compared LASSO, SVR-RBF, and XGBoost, where XGBoost represents a tree-based approach. LASSO achieved the highest prediction accuracy while also requiring the shortest training time, making it the most suitable method for constructing more than 16 million reduced-form models.

The corresponding comparisons have been added to the revised Materials and methods (p. 7, lines 154–164; p. 8, lines 172–187) and Results (pp. 12–13, lines 270–308) sections.

9. Line 156: It is unclear how “little variation” is defined. What is the criterion? If the authors only want to remove ocean cells, that is probably fine. But please be consistent and explicitly list the criteria to avoid potential biases.

Answer) We thank the reviewer for pointing out the need to clearly define the criterion for “little variation.” We agree that explicitly stating the criterion helps avoid ambiguity and potential bias.

In this study, “little variation” was defined as cases where the response variable contained five or fewer unique values in the training data. In such cases, LASSO model training was replaced by a constant predictor equal to the mean of the training responses because reliable model fitting was not possible. This criterion was applied uniformly to all grid cells, days, and hours throughout the simulation domain and was not intended to identify or remove ocean cells or any other specific geographic regions.

We revised the Materials and methods section to explicitly describe this criterion and its rationale (p. 7, lines 156–162). We also added a quantitative evaluation in the Results section and S5 Table, showing that such cases occurred primarily outside the 17 administrative regions and were extremely rare within the primary study area (p. 14, lines 311–320).

10. Lines 188–194: Please explain the meaning of the coefficients from the LASSO models and the meaning of the influence ratio. To my understanding, the predictors are emission scaling ratios. Therefore, it is unclear what it means to sum up the coefficients for scaling factors.

Answer) We thank the reviewer for this valuable comment. As explained in our response to Comment 7, the fitted LASSO coefficients should be interpreted as coefficient-based sensitivities within the surrogate modeling framework rather than as physical source contributions from CMAQ. Because all predictor variables are dimensionless emission scaling factors defined on a common scale, their coefficients can be directly compared and aggregated.

To address the reviewer’s concern, we substantially revised the Materials and methods section by providing explicit mathematical definitions of the influence ratios and added schemat

Attachment Submitted filename: PONE_Rebuttal.docx

# Peer review: Decision Letter 1

**DOI:** 10.1371/journal.pone.0347073.r003

**Authors:** Yangyang Xu

31 Aug 2026

A LASSO-based reduced-form CMAQ model for predicting ozone and PM2.5 responses to emission changes in South Korea

PONE-D-26-15138R1

Dear Dr. Hwang,

We’re pleased to inform you that your manuscript has been judged scientifically suitable for publication and will be formally accepted for publication once it meets all outstanding technical requirements.

Within one week, you’ll receive an e-mail detailing the required amendments. When these have been addressed, you’ll receive a formal acceptance letter and your manuscript will be scheduled for publication.

An invoice will be generated when your article is formally accepted. Please note, if your institution has a publishing partnership with PLOS and your article meets the relevant criteria, all or part of your publication costs will be covered. Please make sure your user information is up-to-date by logging into Editorial Manager at Editorial Manager® and clicking the ‘Update My Information' link at the top of the page. For questions related to billing, please contact billing support.

If your institution or institutions have a press office, please notify them about your upcoming paper to help maximize its impact. If they’ll be preparing press materials, please inform our press team as soon as possible -- no later than 48 hours after receiving the formal acceptance. Your manuscript will remain under strict press embargo until 2 pm Eastern Time on the date of publication. For more information, please contact onepress@plos.org.

Kind regards,

Yangyang Xu

Academic Editor

PLOS One

Additional Editor Comments (optional):

Reviewers' comments:

Reviewer's Responses to Questions

**Comments to the Author**

Reviewer #1: All comments have been addressed

Reviewer #2: (No Response)

**********

2. Is the manuscript technically sound, and do the data support the conclusions?

Reviewer #1: Yes

Reviewer #2: Yes

**********

3. Has the statistical analysis been performed appropriately and rigorously?

Reviewer #1: Yes

Reviewer #2: Yes

**********

4. Have the authors made all data underlying the findings in their manuscript fully available?

The PLOS Data policy

Reviewer #1: Yes

Reviewer #2: Yes

**********

5. Is the manuscript presented in an intelligible fashion and written in standard English?

Reviewer #1: Yes

Reviewer #2: Yes

**********

Reviewer #1: The authors have addressed most of my concerns. I appreciate it. I support publishing this paper in this journal.

Reviewer #2: Thanks for addressing most of my comments. I might have missed it, but could you also include the list of emission variables (which pollutants are considered in the emission inventory)?

**********

what does this mean?). If published, this will include your full peer review and any attached files.

If you choose “no”, your identity will remain anonymous but your review may still be made public.

**Do you want your identity to be public for this peer review?**  For information about this choice, including consent withdrawal, please see our Privacy Policy

Reviewer #1: **Yes:** Yuzhou Wang

Reviewer #2: No

**********

# Editor report: Acceptance letter

**DOI:** 10.1371/journal.pone.0347073.r004

**Authors:** Yangyang Xu

PONE-D-26-15138R1

PLOS One

Dear Dr. Hwang,

I'm pleased to inform you that your manuscript has been deemed suitable for publication in PLOS One. Congratulations! Your manuscript is now being handed over to our production team.

At this stage, our production department will prepare your paper for publication. This includes ensuring the following:

* All references, tables, and figures are properly cited

* All relevant supporting information is included in the manuscript submission,

* There are no issues that prevent the paper from being properly typeset

You will receive further instructions from the production team, including instructions on how to review your proof when it is ready. Please keep in mind that we are working through a large volume of accepted articles, so please give us a few days to review your paper and let you know the next and final steps.

Lastly, if your institution or institutions have a press office, please let them know about your upcoming paper now to help maximize its impact. If they'll be preparing press materials, please inform our press team within the next 48 hours. Your manuscript will remain under strict press embargo until 2 pm Eastern Time on the date of publication. For more information, please contact onepress@plos.org.

You will receive an invoice from PLOS for your publication fee after your manuscript has reached the completed accept phase. If you receive an email requesting payment before acceptance or for any other service, this may be a phishing scheme. Learn how to identify phishing emails and protect your accounts at https://explore.plos.org/phishing.

If we can help with anything else, please email us at customercare@plos.org.

Thank you for submitting your work to PLOS One and supporting open access.

Kind regards,

PLOS One Editorial Office Staff

on behalf of

Dr. Yangyang Xu

Academic Editor

PLOS One
